# Testicular cancer in mice: interplay between stem cells and endocrine insults

**DOI:** 10.1186/s13287-022-02784-5

**Published:** 2022-06-08

**Authors:** Ankita Kaushik, Deepa Bhartiya

**Affiliations:** Stem Cell Biology Department, ICMR-National Institute for Research in Reproductive and Child Health, Jehangir Merwanji Street, Parel, Mumbai, 400 012 India

**Keywords:** Testis, Cancer, Stem cells, VSELs, Cancer stem cells, OCT-4, CD166

## Abstract

**Background:**

Incidence of type II germ cell tumors (T2GCT) has increased in young men possibly due to fetal/perinatal exposure to estrogenic compounds. Three-fold increased incidence of T2GCT was reported in men exposed in utero to diethylstilbestrol (DES). T2GCT is a development-related disease arising due to blocked differentiation of gonocytes into spermatogonia in fetal testes which survive as germ cell neoplasia in situ (GCNIS) and initiate T2GCT. In our earlier study, T2GCT-like features were observed in 9 out of 10 adult, 100-day-old mice testes upon neonatal exposure to DES (2 μg/pup/day on days 1–5). Neonatal DES exposure affected testicular very small embryonic-like stem cells (VSELs) and spermatogonial stem cells and resulted in infertility, reduced sperm counts and tumor-like changes leading to our postulate that testicular dysgenesis syndrome possibly has a stem cell basis. The present study was undertaken to further characterize testicular tumor in mice testes.

**Methods:**

DES-exposed mice pups (*n* = 70) were studied on D100 and after 12 months to understand how T2GCT progresses. Besides histological studies, a carefully selected panel of markers were studied by immuno-fluorescence and qRT-PCR.

**Results:**

DES resulted in either atrophied or highly vascularized, big-sized testes and extra-testicular growth was also observed. GCNIS-like cells with big, vacuolated cytoplasm and increased expression of OCT-4, SSEA-1, SCA-1 and CD166 (cancer stem cells marker) along with reduced c-KIT, MVH and PTEN were evident. Global hypomethylation was found associated with altered expression of Dnmts, Igf2-H19 and Dlk-Meg3 imprinted genes along with reduced expression of Ezh2, cell cycle regulator p57KIP2 and Meg3; however, Pten remained unaltered. Increased expression of PCNA and Ki67 was observed in concert with complete lack of SOX-9 suggesting Sertoli cells independent proliferation.

**Conclusions:**

Mouse model for T2GCT is described which will have immense potential to understand cancer initiation, cancer stem cells and also to develop effective therapies in future. T2GCT initiates from tissue-resident, pluripotent VSELs due to their altered epigenome. Neonatal exposure to DES blocks differentiation (spermatogenesis) and VSELs get transformed into CD166 positive cancer stem cells that undergo excessive self-renewal and initiate cancer in adult life challenging existing concept of fetal origin of T2GCT.

**Supplementary Information:**

The online version contains supplementary material available at 10.1186/s13287-022-02784-5.

## Introduction

Type II germ cell tumors (T2GCT) account for only 1% of all malignancies in males but are the most common solid tumors, occurring in young men (18–35 years), and represent the leading cause of cancer-related deaths. Incidence of T2GCT has increased 65% during the course of the last 40 years due to perinatal exposure to estrogenic compounds [1]. Testicular cancer is generally considered to have a fetal origin, gonocytes fail to differentiate into spermatogonia upon exposure to endocrine disruptors and retain their pluripotency [2, 3]. These cells or GCNIS (germ cell neoplasia in situ) are germ cells blocked in development and upon gaining chromosomal anomalies over time possibly initiate T2GCT in young men [4, 5]. Gene expression profile of GCNIS and fetal germ cells is very similar and both express markers like PLAP, KIT, OCT3/4 and Ki-67 suggesting that T2GCT/GCNIS arise from the fetal gonocytes [6, 7]. Prospective clinical studies have proved difficult due to rare nature on T2GCT and only few exposed to any kind of perinatal insults may develop it over time. A recent meta-analysis by Hom et al. [8] showed a three-fold increase in testicular cancer risk among men who were exposed in utero to diethylstilbestrol (DES), implicating early hormonal exposures in etiology of testicular cancer. There is no mice model for T2GCT at present and this makes the field very challenging [2].

Testicular stem cells include two distinct populations including very small embryonic-like stem cells (VSELs) and spermatogonial stem cells (SSCs) in both human and mice testes, and a robust protocol was described recently to enrich them after enzymatic digestion of testicular tissue [9–12]. They express gonadotropin (FSHR) and steroid (Erα and Erβ) hormone receptors and thus are vulnerable to endocrine disruption [11, 13, 14]. We recently reported that endocrine disruption during neonatal life affected stem cells in mice testes and blocked differentiation (spermatogenesis) of VSELs/SSCs that results in reduced sperm count and sub-fertility/infertility whereas excessive self-renewal of pluripotent VSELs results in cancer-like changes in mice testes during adult life [13, 14]. Thus, rather than the fetal gonocytes surviving as a GCNIS in adult testes that results in T2GCT, tissue-resident stem cells possibly initiate testicular cancer in adult life as discussed earlier [15]. Gonocytes, T2GCT as well as VSELs express similar pluripotent markers including OCT-4, NANOG, PLAP, etc. VSELs have recently been reviewed Ratajczak et al. [16] and their altered differentiation could initiate cancer [17].

 Both somatic and germ cells in the neonatal testes get exposed and are affected by DES but somatic cells have a finite life span and are continuously replaced, whereas VSELs are immortal and are the only cells that have the potential to carry the perinatal defects to result in pathologies in adult life. Treatment with DES (2 µg/day/pup for 1–5 days) led to testicular cancer-like changes in 9 of 10 100-day-old adult mice [13]. Flow cytometry studies showed that VSELs (2–6 µm, 7AAD-viable cells with surface phenotype LIN-CD45-SCA-1 +) increased more than seven-fold in numbers along with upregulation of transcripts specific for pluripotent markers *Oct-4A* (*˃*eight-fold), *Sox-2 & Nanog* (40-fold), *Sca-1* (21-fold) and *Oct-4* (12-fold) by qRT-PCR. This increase in VSELs numbers and pluripotent transcripts was associated with blocked meiosis and further differentiation as evident by reduction in 4n population (pachytene spermatocytes) during ploidy analysis, five-fold reduction in c-KIT positive cells by flow cytometry, significant reduction of transcripts specific for *c-Kit* and meiotic marker *Scp-3* by qRT-PCR [13].

VSELs are quiescent in nature and do not divide readily in vitro nor form teratoma because of their unique DNA methylation pattern at some developmentally crucial, imprinted genes [15, 18]. It is likely that epigenetic changes in the VSELs by neonatal exposure to endocrine disruption result in adult onset of various pathologies. The present study was undertaken to further characterize the testicular cancer-like changes induced by neonatal exposure to DES in 100-day and ˃12-month-old mice. Besides histological changes, expression of stem cells (OCT-4A, OCT-4, SSEA-1, SCA-1), early differentiation (c-KIT, MVH, Dmrt1a), Sertoli cells (SOX9), cancer stem cells (CD166), proliferation (PCNA, Ki67), global methylation (5mC) and tumor suppresser (PTEN)-specific markers were studied. Also, transcripts specific for estrogen receptors (*Esr-1, Esr-2),* alternately spliced *Fshr isoforms* (*Fshr-1, Fshr3*), imprinted genes (*Igf2, H19, Dlk, Meg3*), tumor suppressor gene (*Pten),* cyclin-dependent kinase inhibitor (*p57kip2), Ezh2* and DNA methylation enzymes (*Dnmt1, Dnmt3a, Dnmt3b, Dnmt3L*) were studied by qRT-PCR. Reasons for selecting this panel of markers for the present study are listed in Additional file 1: Table S1 and will hopefully provide a bird’s eye view of the effect of endocrine disruption on the VSELs epigenome.

## Study design and methods

Adult Swiss mice, maintained in the Institute Experimental Animal Facility, were used for the present study. The animals were housed under controlled temperature of (23 ± 1 °C) and humidity (55 ± 5%), with 14 h light/10 h dark cycle with free access to food and water. All experiments carried out in the present study were approved by Institutional Animal Ethics Committee. Mice pups were administered DES (2 μg/pup/day on days 1–5, subcutaneous route). Control mice received vehicle (sesame oil) alone. They were killed after 100 days and ˃12 months of exposure to DES.

### Histology

Testes were removed, weighed and then fixed in 4% paraformaldehyde (Sigma) for histological studies. Paraffin blocks were prepared and 5-µm sections were cut using standard methods. The sections were deparaffinized and stained with Hematoxylin and Eosin using standard methods. The slides were viewed under microscope and the representative fields were photographed using Nikon bright-field microscope (90i NIKON).

### Immuno-localization studies

Paraffin-embedded tissue sections were used for immuno-localization studies to study expression of VSELs (OCT-4A, SSEA-1), spermatogonial stem cells (SCA-1, OCT-4), germ cells (c-KIT, MVH), Sertoli cells (SOX-9), proliferation (Ki-67, PCNA), tumor suppressor marker (PTEN), 5-Methyl Cytosine (5mC) for global methylation and Activated Leukocyte Cell Adhesion Molecule (ALCAM/CD166) as a cancer stem cells marker. The details of the antibodies used in the present study are provided in Additional file 1: Table S2. The sections were deparaffinized by immersing the slides in xylene 2 times for 15 min each followed by their rehydration through a graded methanol series (100%, 70%, 50% and 30%). Unmasking of the epitopes was done by antigen retrieval by treating the sections with sodium citrate buffer (10 mM sodium citrate, pH 6.0) at high power for 8 min in a microwave oven. The sections were permeabilized using 0.5% Triton-X 100 for 5 min only to study nuclear expression of OCT-4A, PCNA and Ki67. This step was avoided for the cell surface and cytoplasmic antigens. Sections were labeled with a hydrophobic barrier pen.

Blocking of the non-specific sites was done with 5% goat serum (NGS) and 1% BSA for 1 h followed by incubation with the primary antibody at 4C overnight. Following incubation of primary antibody, the slides were washed 3 times for 5 min each in 0.5% BSA in PBS (wash buffer) and incubated with fluorescent tagged secondary antibody (Alexafluor-488 or Alexafluor-568) for 2 h at room temperature in dark. The slides were washed 3 times for 5 min each in wash buffer and counterstained with DAPI for 20 min. Excess wash buffer was removed and sections were cover slipped using Vectastain mounting medium. Edges of the coverslips were sealed with nail paint to prevent the cells from drying. Later the slides were examined under laser scanning confocal microscope (OLYMPUS FLUOVIEW FV3000) at 60 × magnification. Negative controls were always run in all the studies with the omission of primary antibody. Representative images are shown in Additional file 1: Fig S1. 

### Quantitative RT-PCR studies

Testicular tissue was placed in TRIzol (Invitrogen) and stored at − 80 °C for RNA isolation. Total RNA was extracted using standard protocol using TRIzol and treated with DNase I (Fermentas, USA) to remove any genomic DNA present. First-strand cDNA was synthesized using the iScript cDNA synthesis Kit (Bio-Rad, USA) according to the manufacturer’s instructions. Briefly, 1 μg of total RNA was incubated with 5× iScript reaction mix and reverse transcriptase mix. The reaction was carried out in G-STORM thermocycler (Gene Technologies, UK) as per manufacturer’s instructions. The expression levels of various gene transcripts were estimated by CFX96 real-time PCR system (Bio-Rad Laboratories, USA) using SYBR Green chemistry (Bio-Rad). 18S was used as housekeeping in mice experiments. The primers used in the study are mentioned in Additional file 1: Table S3. The amplification conditions were: initial denaturation at 94 °C for 3 min followed by 40 cycles comprising of denaturation at 94 °C for 10 s, annealing for 20 s, and extension at 72 °C for 30 s followed by melt curve analysis. The fluorescence emitted was collected during the extension step of each cycle. The homogeneity of the PCR amplicons was verified by running the products on 2% agarose gels and also by studying the melt curve. All PCR amplifications were carried out in duplicate. Mean Ct values generated in each experiment using the CFX Manager software (Bio-Rad) were used to calculate the mRNA expression levels. Since ΔCt is inversely proportional to relative mRNA expression levels, the levels were calculated manually by the ΔCt method. The fold change was calculated using ΔΔCt method. The relative expression level was represented as the fold difference to the value of control taken as 1 and from at least four independent experiments performed on age matched vehicle-treated control and DES exposed group.

### Statistical analysis

Arithmetic means and standard error values of data were calculated using MS Excel. Data were analyzed using Student’s t test for unpaired samples. Statistical significance was defined as *P* < 0.05 and error bars in graphs represent ± SE.

## Results

Histological changes on D100 upon neonatal exposure to DES treatment were briefly described earlier. Spermatogenesis was disrupted and was associated with reduced 4n cells population by ploidy studies and numbers of tubules in stage VIII [13]. In the present study, detailed follow-up studies were undertaken and mice were examined at D100 and after 12 months of age for testicular changes due to neonatal exposure to DES.

### Effect of neonatal exposure to DES on testes gross appearance and weight

**Fig. 1:**
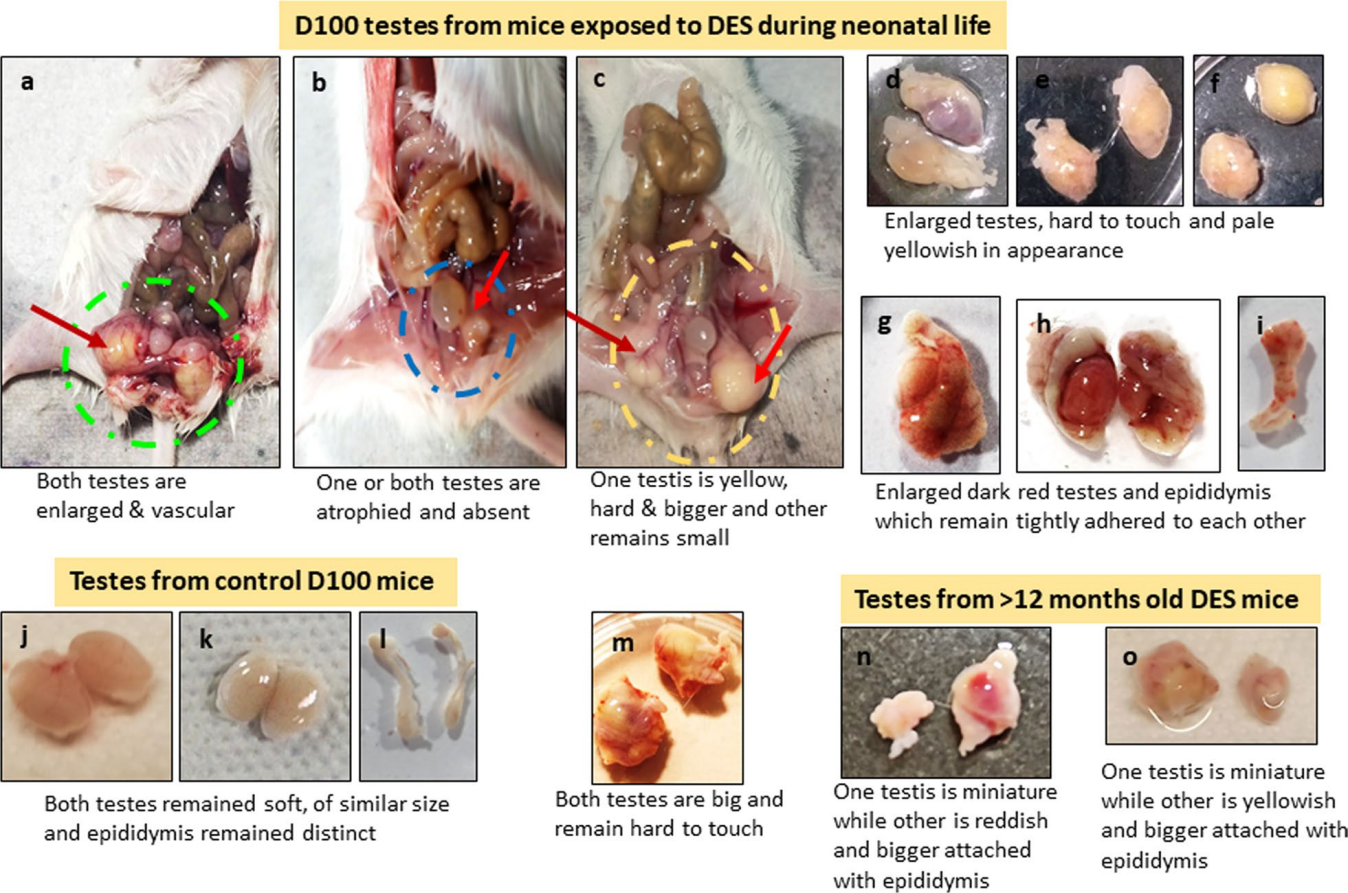
Effect of DES treatment on mice testes. DES exposure led to wide range of abnormal changes. Vehicle-treated control testes were soft and oval shaped (**j**, **k**). In few mice, testes were atrophied (**b**) and in others they were bigger in size (**a**, **d**, **e**) red in colored (**g**, **h**) and very hard to touch (**d**–**f**, **m**). At places, epididymis was tightly adhered to testis (**g**, **h**). Epididymis was reddish (**i**) and completely different from control (**l**). Aged DES-exposed mice testes also had pathology (**n**, **o**)

Seventy DES-treated mice at D100 were used for the study. Gross appearance of testes in mice exposed neonatally to DES showed lot of variation at the time of sacrifice. Of these, 28 had enlarged reddish testes completely adhered to epididymis (Fig. 1a, g, h). Eighteen mice had testes which were pale yellow and big, round in shape and hard to touch (Fig. 1d–h). One of the two testes was big and other was atrophied in 5 mice (Fig. 1c), 3 showed one miniature testis while the other had completely disappeared (Fig. 1b), whereas in 2 mice, both testes were atrophied and not visualized. Testes appeared normal in four mice but all showed affected spermatogenesis. Twelve aged mice (˃12 months of age) neonatally exposed to DES were also studied. Six of them showed enlarged testes. Two mice had atrophied testes, whereas in 4 one testis was big and other was small. In one D100 mouse, a big extra-testicular growth was observed in the pelvic region (Additional file 1: Table S4). Compared to these variations in DES-treated testes, testes in normal age matched control mice remained soft, oval in shape, of similar size and with distinctly separated epididymis (Fig. 1j–l). Relative testicular weights were reduced in atrophied testes and increased in tumor samples (Additional file 1: Fig S2). Death of 14 mice was noted in DES-treated group but the cause of death was not ascertained.

### Histological studies

#### DES-treated atrophied testes on D100

**Fig. 2:**
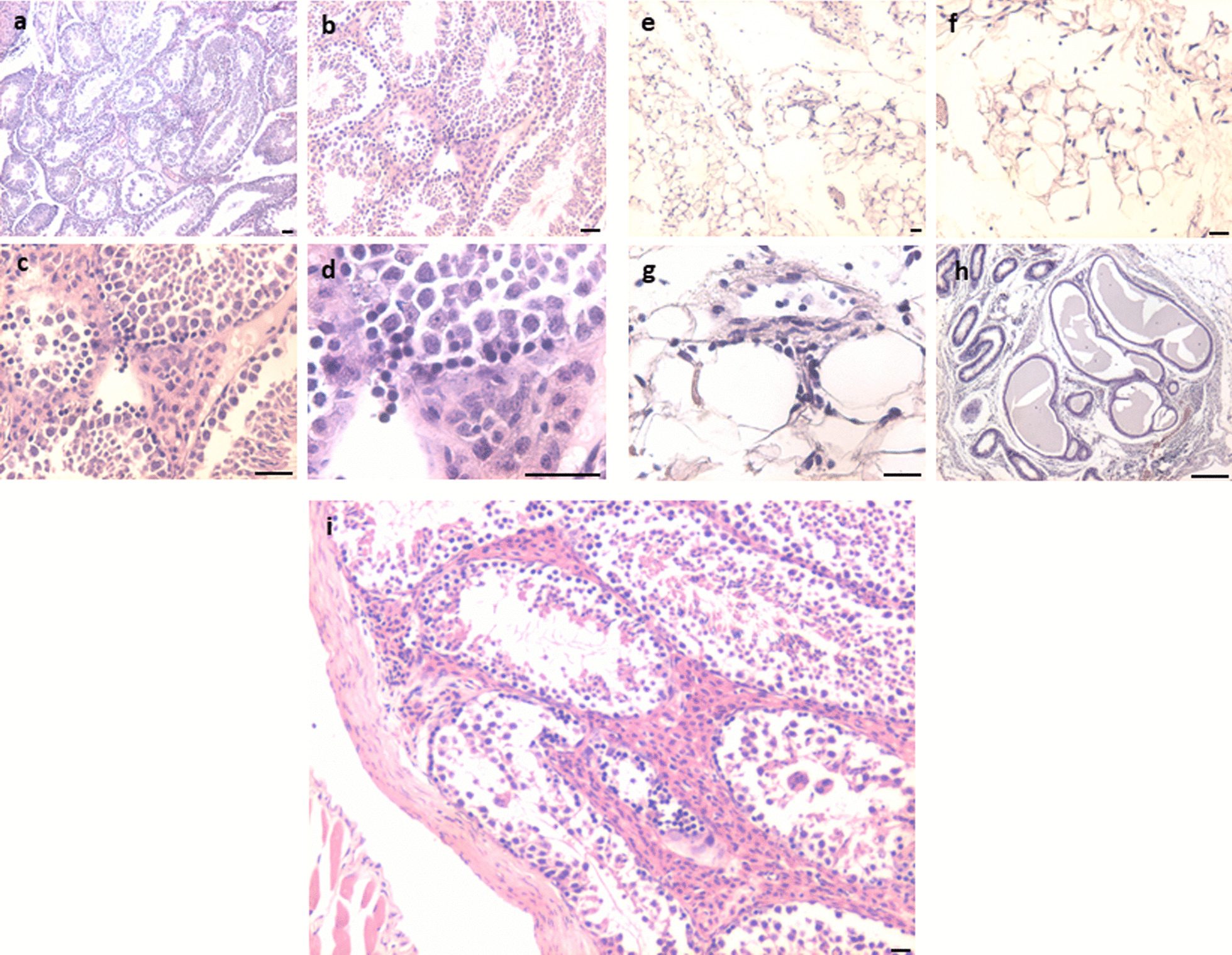
Neonatal exposure to DES resulted in small-sized atrophied testes. Atrophied testes showed reduced spermatogenesis. The seminiferous tubules were empty and with a dilated lumen (**a**, **b**). Spermatid retention was observed (**b**, **i**). Spermatids are pulled down to basal Sertoli cells cytoplasm (asterisk). Darkly stained, spermatogonial cells were prominent along the basement membrane (**i**). Complete histo-architecture was lost (**e**, **f**) and there were no germ cells in seminiferous tubules (**g**). Epididymis was completely devoid of sperm (**h**). Disordered germ cells arrangement was observed. Interstitial hyperplasia was prominent (**c**, **d**) and giant cells were observed at places (arrow). These cells are formed due to germ cell degeneration. Scale: 20 µm

The presence of testicular atrophy increases the risk of bilateral neoplasia considerably [19]. Atrophied testes showed complete loss of testicular histo-architecture. Tubules were present but disorganized spermatogenic cell arrangement with reduced spermatogenesis (Fig. 1a, b) was observed, and small spherical dark stained, putative spermatogonial cells were clearly visualized in chains. Dilated lumen was seen and hyperplasia of interstitial cells was clearly evident. The epididymis also showed complete lack of sperm (Fig. 2g, h). These observations suggested that differentiation was blocked and there was lot of activity in the stem cells compartment. Giant undifferentiated germ cells were evident.

#### DES-treated tumor-like testes on D100

**Fig. 3:**
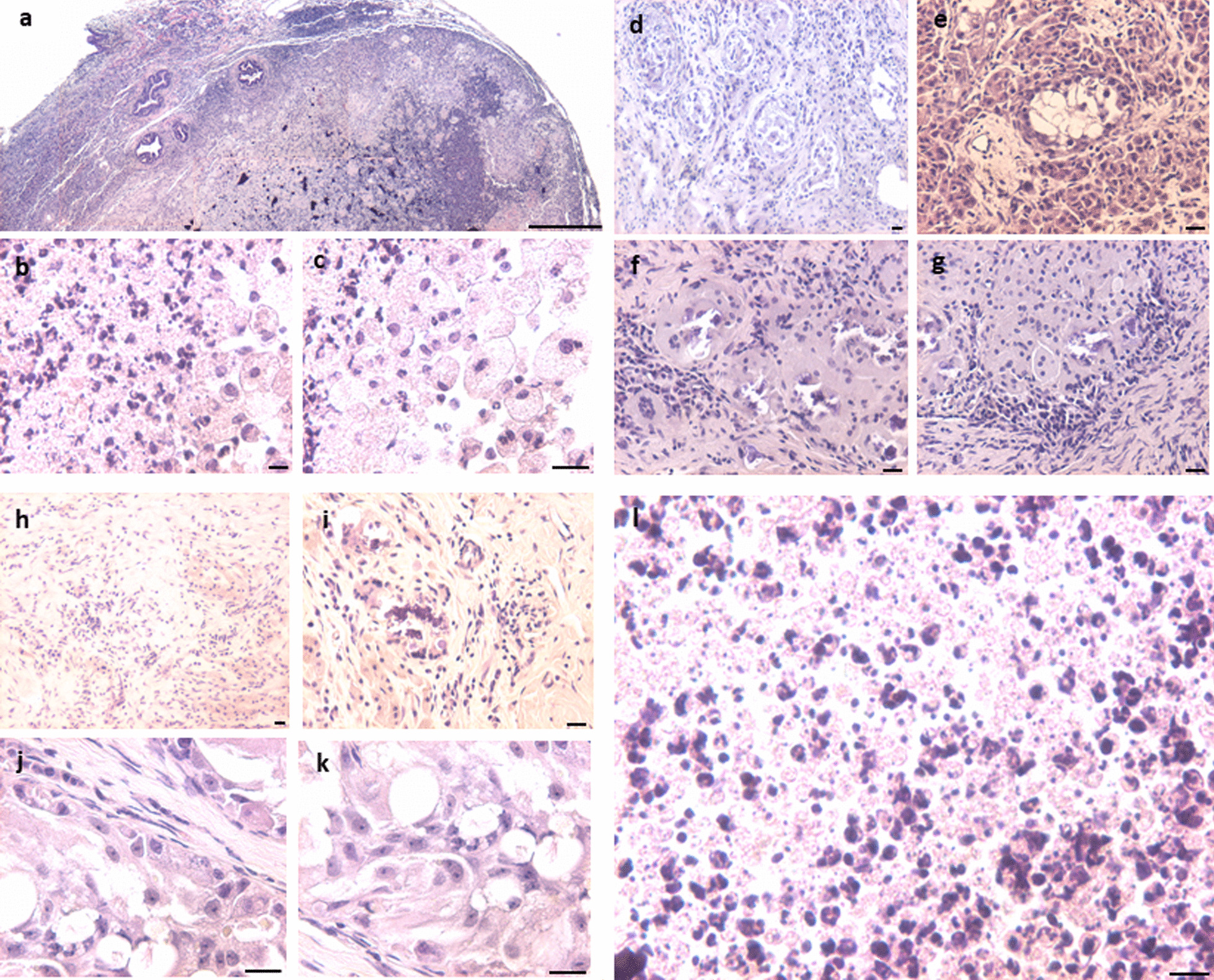
Neonatal exposure to DES resulted in bigger size testes. Bigger testis showed completely disrupted histo-architecture (**a**). Seminiferous tubules were significantly reduced without defined membrane and germ cells were depleted (**d**, **e**). Sertoli cells were also not observed (**d**–**k**). Massive inflammation was observed in testes (**i**). Degeneration of germ cells was observed. GCNIS like stem cells (arrow) were clearly observed restricted to small seminiferous tubule (**d**, **e**, **f**, **g**). Small, spherical ‘putative stem cells’ were present among the inflammatory cells (**h**, **i**, **l**). Multinuclear giant cells were observed (**b**, **c**). Scale: (a:100  µm, k-l: 20 µm)

Big-sized testes showed massive inflammation and complete loss of tubules (Additional file 1: Figs. S3, S4). Gross histology showed complete distorted testes (Additional file 1: Fig. S3). Giant cells were observed at places resembling GCNIS as described in the literature with abundant cytoplasm (Fig. 3d, e). Atypical germ-like cells were observed at places (Additional file 1: Fig. S4). What was of interest was the presence of small, spherical darkly stained cells among the inflammatory cells (Fig. 3i). Epididymis was devoid of sperm and at places germ cells sloughing was observed in the lumen of seminiferous tubules as well as in the epididymal tubular sections. Germ cells appeared to be sloughed off into the lumen. At places the tubular lumen was filled with the inflammatory cells (Fig. 3i).

#### Extra-testicular tumor-like growth

**Fig. 4:**
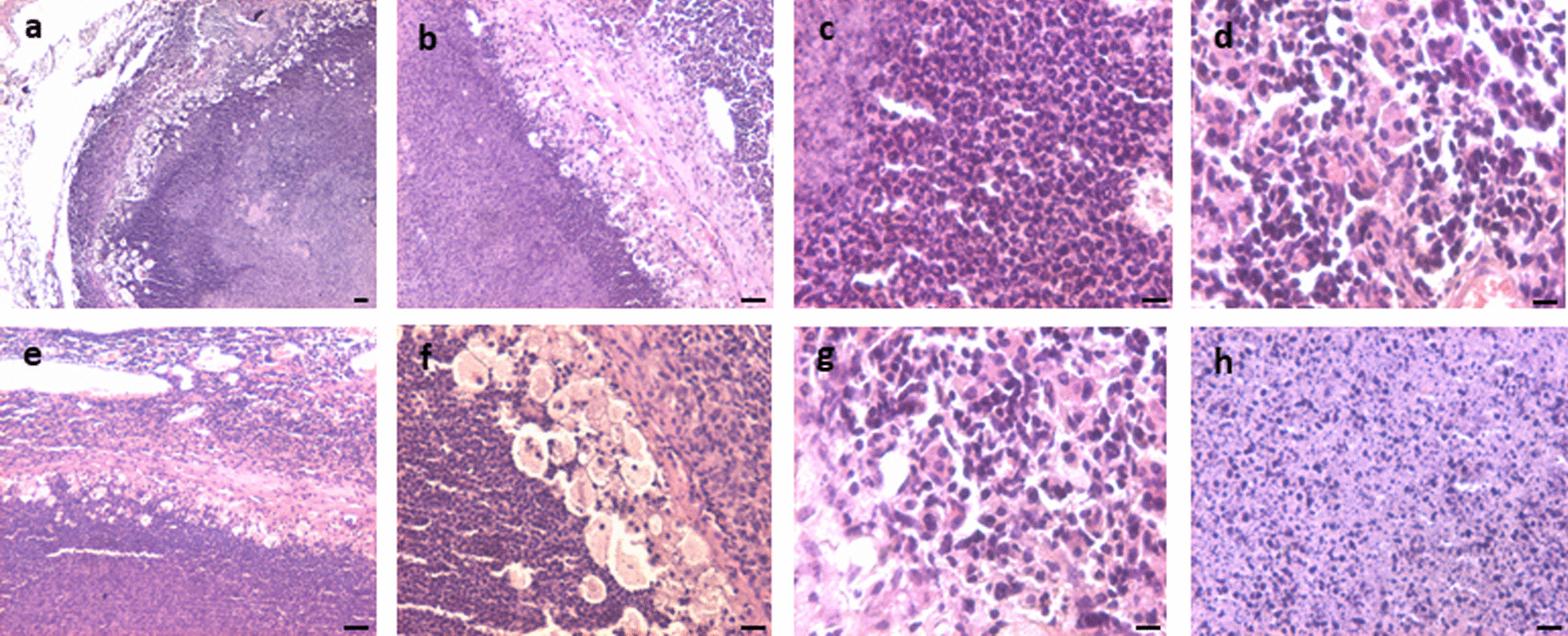
Histological features of extra-testicular growth. The central region was filled with inflammatory cells (**a**–**f**, **h**), then there was a ring of giant cells (**f**) with highly vacuolated cytoplasm and on the outer surface, large numbers of cells were present (**f**, **g**). Scale: 20 µm

In one mouse, a pea-size extra-testicular growth was observed in between the two testes. It was highly vascular and upon sectioning, highly cellular structure was observed. There was a layer of inflammatory cells, surrounded by large numbers of giant, oval cells (GCNIS) toward the outside. Besides inflammatory cells which were identified with multilobed nuclei, large numbers of spherical cells were observed with dark stained nuclei and high nucleo-cytoplasmic ratio. Increased cellularity was observed with multinucleated cells (Fig. 4d).

#### Control testes

**Fig. 5:**
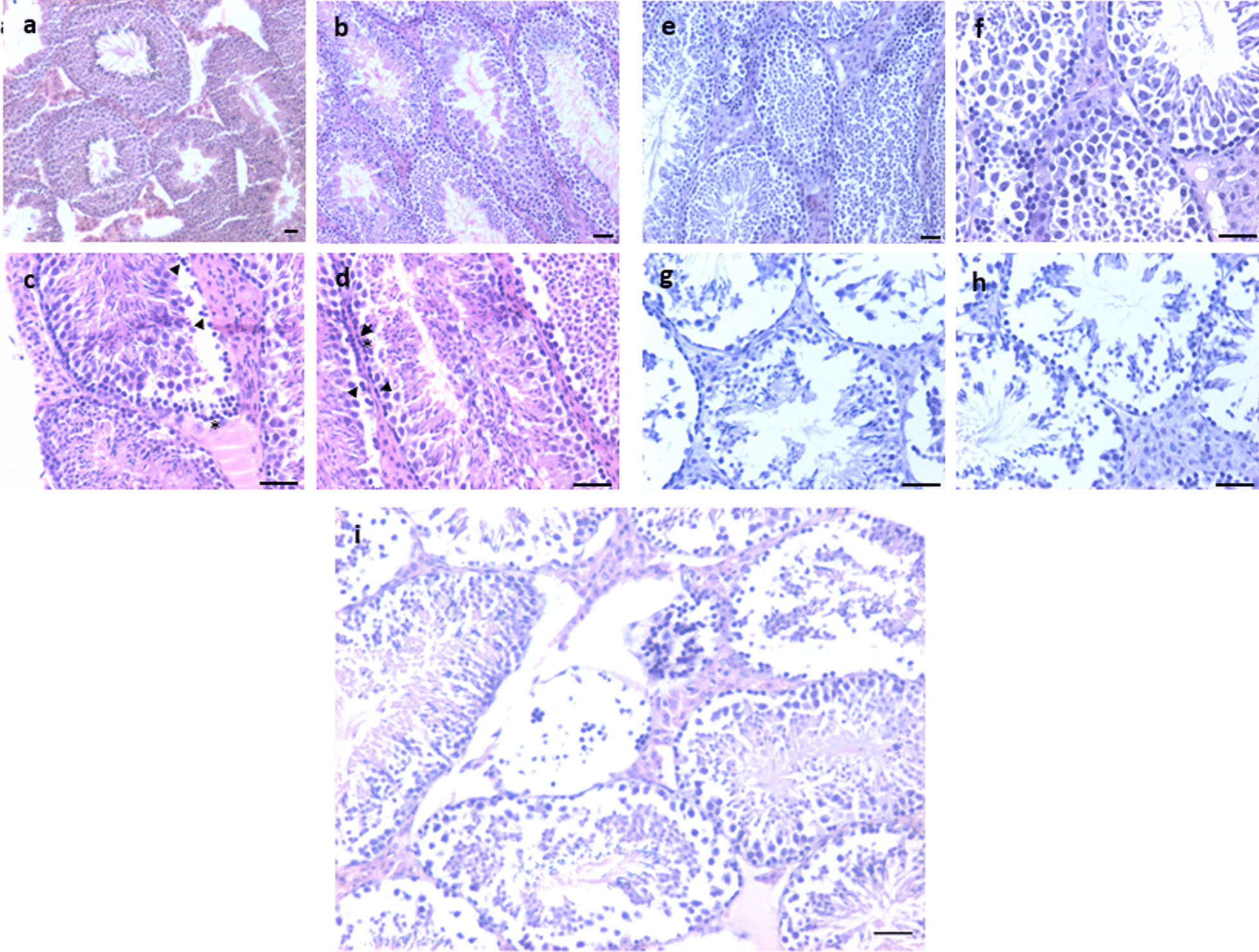
Testicular section of 100 days old adult mice showed normal spermatogenesis. Tubules had well-defined basement membrane and lumen was filled with sperm. Spermatogenic and Sertoli cells were clearly visible (**a**). Aged (>12 months old) mice showed testicular atrophy and suppressed spermatogenesis (**b**–**i**). Significant hyperplasia of the interstitial compartment is evident (**f**). Tubules were tightly packed with disrupted spermatogenesis (**b**, **e**, **g**–**i**). Small=sized, darkly stained, spherical cells were evident along the basement membrane of the tubules (**c**–**f**). At places they were seen as doublets or chains (**c**, **d**). The balance between proliferation and differentiation was affected possibly because the niche provided by the Sertoli cells gets affected with age and thus is unable to support spermatogenesis efficiently [20]. Scale: 20 μm

Testicular sections of 100 days old adult mice showed normal spermatogenesis. Seminiferous tubules were surrounded by a well-defined basement membrane and lumen was filled with sperm. Spermatogenic cells and Sertoli cells were clearly evident (Fig. 5a). Aged, > 12-month-old mice showed testicular atrophy and suppressed spermatogenesis (Fig. 5b–i). Significant hyperplasia of the interstitial compartment was clearly evident (Fig. 5f). Tubules were tightly packed with disrupted spermatogenesis (Fig. 5b, e, g–i). Small-sized, darkly stained, spherical cells were evident along the basement membrane of the tubules (Fig. 5c–f). At places they were seen as doublets or in chains (Fig. 5c–d). The balance between proliferation and differentiation of stem/progenitor cells was clearly tilted toward proliferation and differentiation of germ cells was affected possibly because the niche provided by the Sertoli cells gets affected with age and thus is unable to support spermatogenesis efficiently [20].

#### Aged testes neonatally exposed to DES

**Fig. 6:**
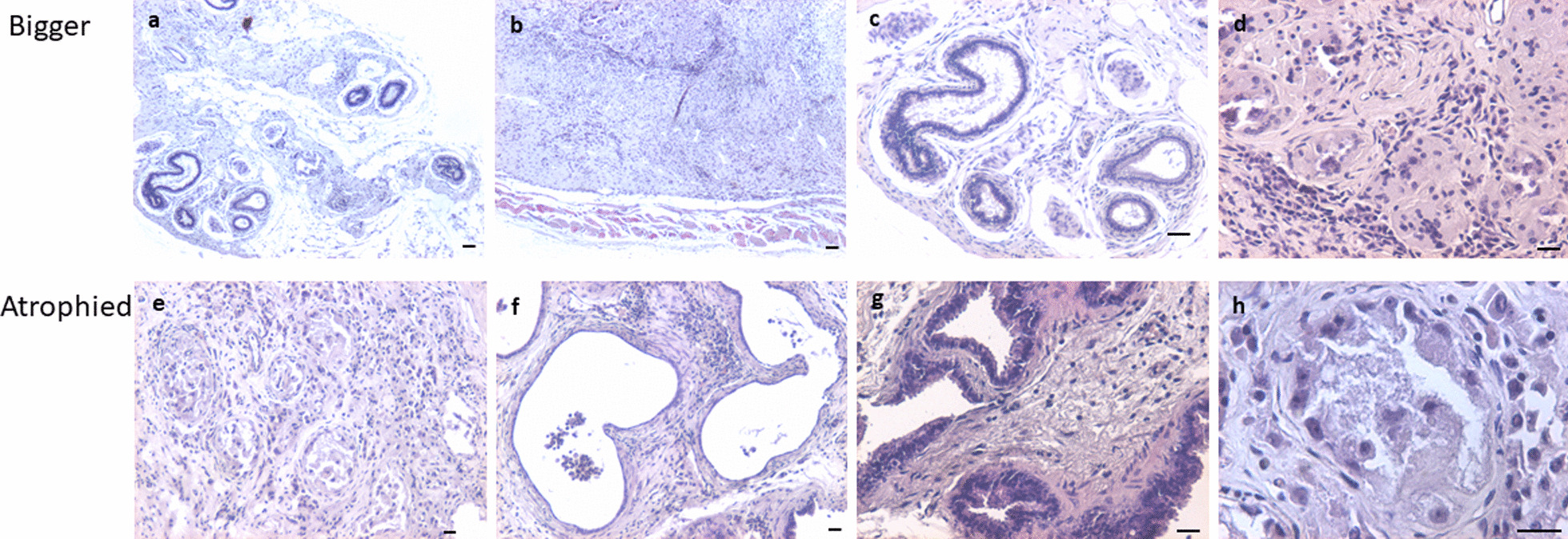
Testicular sections of aged (>12 months old) mice neonatally exposed to DES (**a**–**h**). The testes showed big tumor-like growth (**a**–**d**) and in few mice it appeared atrophied (**e**–**h**). There were no signs of spermatogenesis. Well-defined seminiferous tubules were lacking and proliferation of undifferentiated GCNIS-like atypical, enlarged cells (**h**, arrows) with big nuclei was observed (**e**–**h**) at places. Scale: 20 μm

Aged mice neonatally exposed to DES showed both bigger (Fig. 6a–d) and atrophied testis (Fig. 6e–h). Irregular proliferation of germ cells-like atypical cells with clear nucleoli and massive necrotic changes was observed (Fig. 6e, h). Seminiferous tubules were markedly reduced in numbers and GCNIS cells were clearly observed (Fig. 6h). Increased cellularity was observed in the testicular sections.

### Immunofluorescence studies

**Fig. 7:**
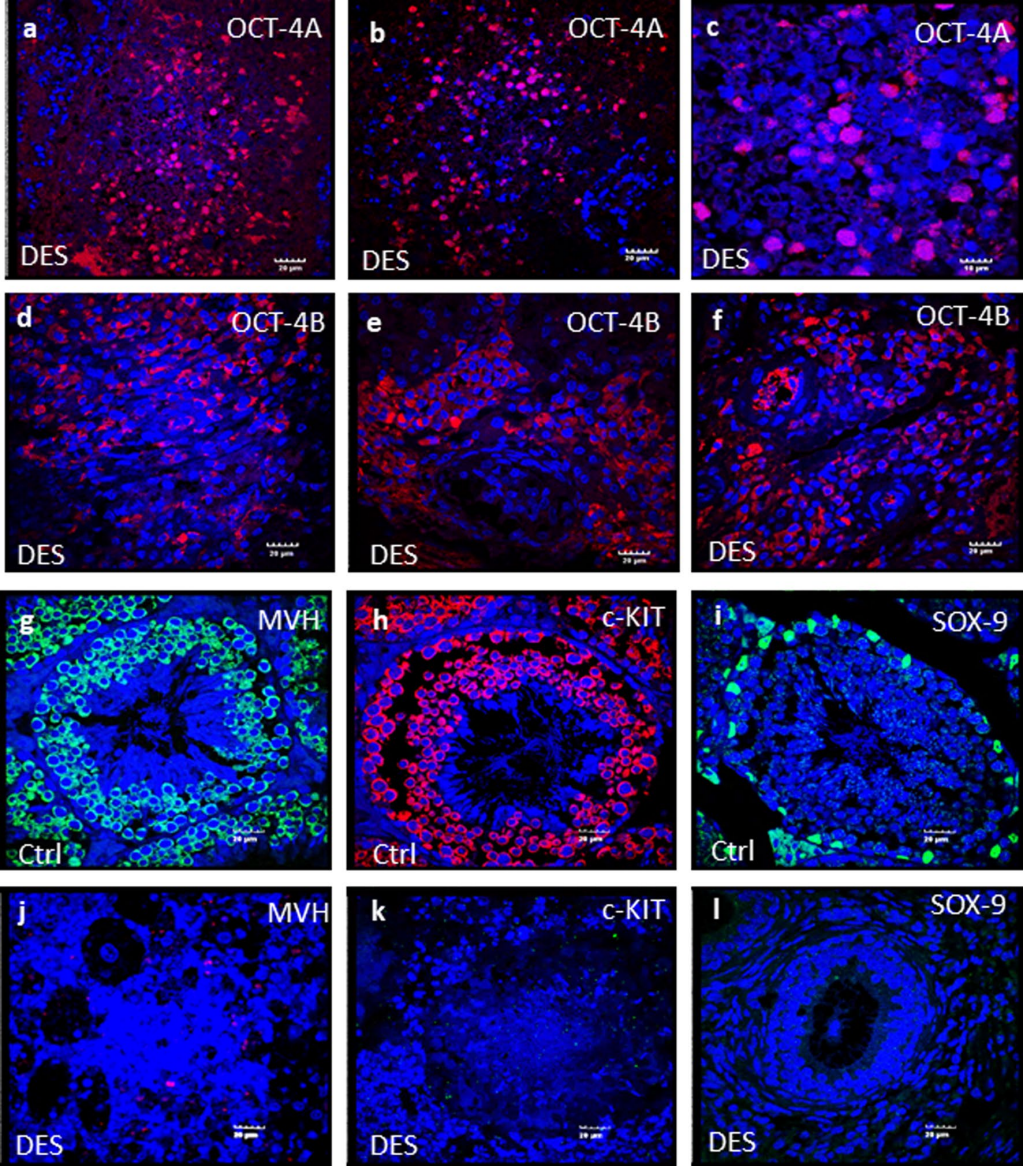
Characterization of tumor-like changes induced by neonatal exposure to DES in adult mice testes were studied by immuno-localization. Note increased expression of VSELs specific nuclear OCT-4A (**a**–**c**) and cytoplasmic OCT-4 (**d**–**f**) after endocrine disruption with DES. MVH (**g**), c-KIT (**h**) positive cells were arranged multi-layered in normal control mice conversely DES-treated testes have no MVH (**j**) and c-KIT (**k**) positive cells. SOX-9 positive Sertoli cells were located at basement of germinal epithelium in control mice (**i**) whereas DES testes are completely devoid of nutritive Sertoli cells (**l**). Results suggests blocked differentiation of pluripotent stem cells but OCT-4 positive stem cell compartment is stimulated in absence of microenvironment upon DES treatment. DAPI staining was performed to visualize the nucleus and the image merged with DAPI (merged) shown here. Scale: 20 µm (**a**–**l**) except (**c**) 10 µm

Increased OCT-4 expression was reported upon neonatal expression to DES by both flow cytometry and qRT-PCR studies [13]. OCT-4A is an indispensable bio-marker for testicular tumor [21, 22] and was found over-expressed in the nuclei of VSELs and in the cytoplasm of bigger spermatogonial stem cells (Fig. 7a–c). It was minimally expressed in D100 control testes (data not shown). These OCT-4 positive cells were observed distributed throughout the testicular stroma and not limited to expression within the seminiferous tubules (were mostly not observed).

MVH was specifically expressed in cytoplasm of germ cells (spermatogonia, spermatocytes and round spermatids) in vehicle-treated control testes (Fig. 7g) and was not detected in DES-treated testis (Fig. 7j). c-KIT is a marker for differentiating spermatogonia and significantly reduced expression was observed in DES-treated testes (Fig. 7k). However, at places few c-KIT positive cells were observed (data not shown). SOX-9, a marker for Sertoli cells, was not observed in DES-treated testes (Fig. 7i) suggesting disrupted testicular microenvironment. Bright-field and DAPI images are shown in Additional file 1: Fig. S5.

**Fig. 8:**
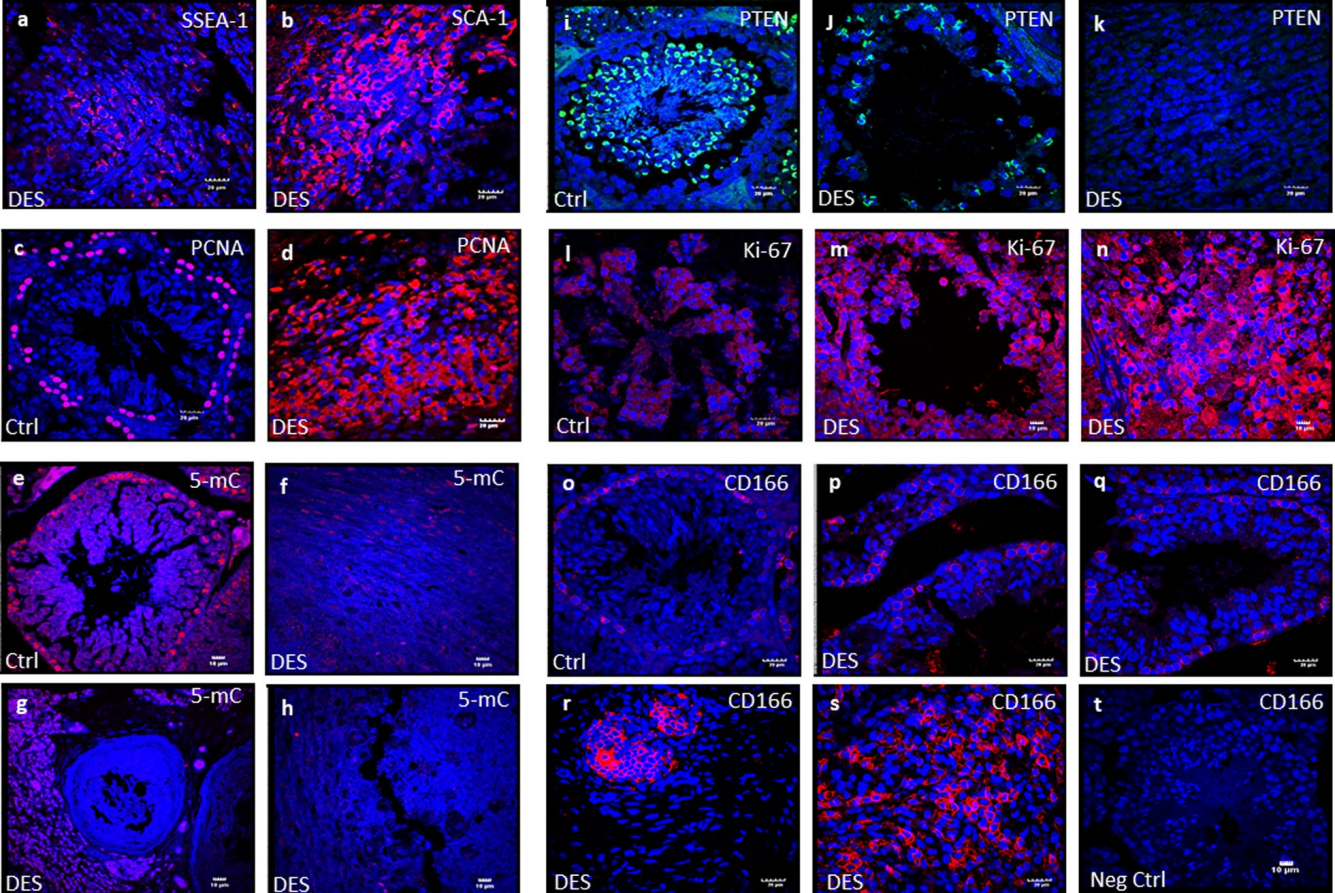
Immuno-localization of SSEA-1, SCA-1, PCNA, Ki-67, PTEN, 5-mC and CD166. SSEA-1 (**a**) and SCA-1 (**b**) positive clearly evident and PCNA (**d**) expression was massively increased in DES-treated testes compared with control (**c**). 5-mC was expressed in spermatogonia along the basement membrane and in spermatocytes in control testes (**e**) but was minimally expressed (**f**), completely loss (**h**) and also some positive cells are also observed at places (**g**) after DES treatment suggestive of global hypomethylation. PTEN was observed germ cells in control testis (**i**) whereas in atrophied testis it was minimally expressed (**j**) but it is completely absent in bigger testis (**k**) suggestive from conducive environment for tumor formation. Marked increased in Ki-67 was observed after DES treatment (**m**, **n**) compared with normal age-matched vehicle-treated control (**l**). CD166 -a cell surface marker for stem/progenitor cells in several cancers was minimally expressed in vehicle-treated control (**o**) and atrophies testis (**p**, **q**), however, massively expressed in bigger testis (r-s) with tumor-like changes. Negative control with omission of primary antibody (**t**). Scale: 20 µm (**a**–**d**, **i**–**k**, **l**, **o**–**s**) and for (**e**–**h** and **m**, **n**, **t**) 10 µm

Increased expression of other stem cell markers SCA-1 (Fig. 8b) and SSEA-1 (Fig. 8a) was also observed in the DES-treated testicular sections. Expression of 5-mC compared to vehicle-treated control testes showed marked reduction (Fig. 8f–h) suggestive of global hypomethylation. Expression of PTEN, a tumor suppressor marker, widely expressed in germinal epithelium (spermatogonia, spermatocytes and round spermatids) in normal testis (Fig. 8i), was studied. PTEN showed loss (Fig. 8k) or minimal expression in DES-treated testes (Fig. 8j). Bright-field and DAPI images are shown in Additional file 1: Fig. S6.

Overexpression of both PCNA (Fig. 8d) and Ki67 (Fig. 8m, n) was observed in DES-treated testes compared to vehicle-treated control. Ki67 was majorly observed on the cell surface and in the cytoplasm rather than the expected nuclear expression and similar expression is earlier reported in breast tumor sections [23]. CD166 expression yielded interesting results. It remained minimally expressed in few cells located in the basal region of the tubules in control group (Fig. 8o). CD166, a marker for the cancer stem cells [24–26], was minimally detected in testicular sections of atrophied testes (Fig. 8p, q) but the bigger, reddish tumor-like testicular sections showed increased expression of CD166 (Fig. 8r, s). Its cytoplasmic or membranous staining pattern is suggestive of progression and metastasis of tumor [27]. Hence, its overexpression in neonatally DES-exposed mice strongly correlated with TGCT progression. Bright-field and DAPI images are shown in Additional file 1: Fig. S7. Such molecular changes were not observed upon neonatal exposure to estradiol (Additional file 1: Fig. S9).

**Fig. 9:**
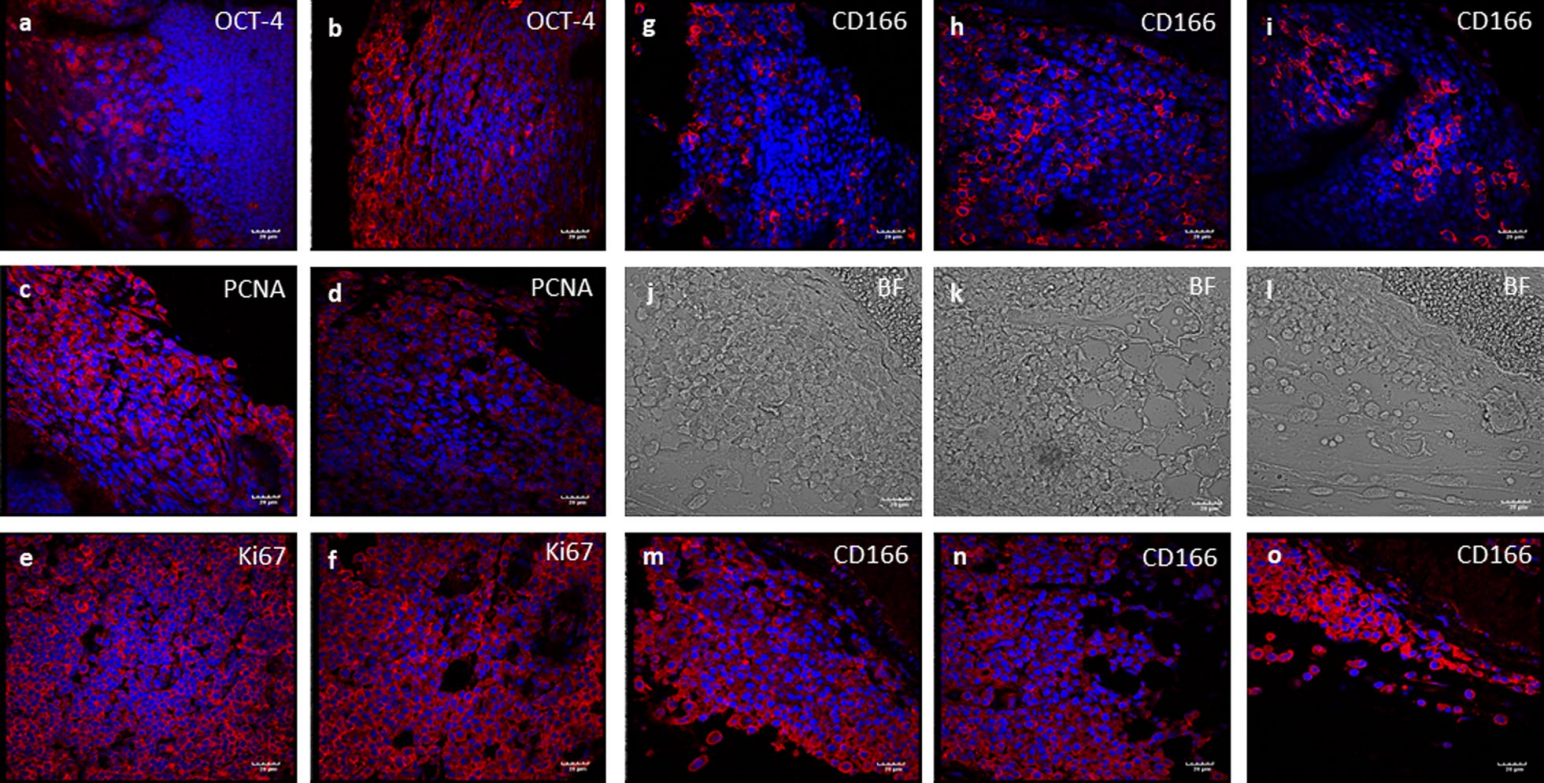
Increased expression of OCT-4, PCNA and Ki-67 was observed in extragonadal tissue. OCT-4 expression was increased (**a**, **b**) along with increased proliferation as evident by expression of PCNA (**c**, **d**) and Ki-67 (**e**, **f**). CD166 positive cells were seen in increased numbers (**g**–**o**) and they are detaching from surface to metastasize at other places (**m**–**o**). Scale: 20 µm

The cells in the extra-testicular growth showed increased expression of OCT4 (Fig. 9a, b), PCNA (Fig. 9c, d), Ki67 (Fig. 9e, f), and also CD166 (Fig. 9g–o). Cells expressing CD166 appeared to be sloughed off from the surface (Fig. 9m–o) into the visceral/ peritoneal cavity. Bright-field and DAPI images are shown in Additional file 1: Fig. S8.

### qRT-PCR studies

**Fig. 10:**
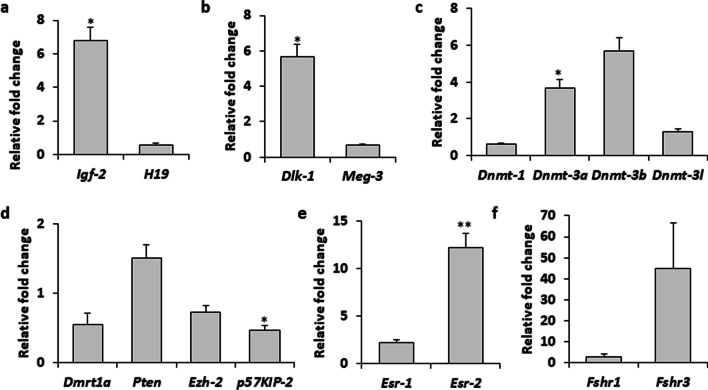
Neonatal exposure to DES treatment affected paternally imprinted genes IGF2-H19 (**a**) and DLK-MEG-3 (**b**). DNA methylation enzymes expression was dysregulated (**c**) and transcripts specific to tumor suppressor (Pten) was not affected (**d**). Dmrt1, Ezh-2 and cell cycle inhibitor specific transcript p57kip-2 was downregulated (**d**). Erβ was increased sevenfold (**e**) and it was interesting to note that alternately spliced Fshr3 is > 40-fold upregulated in DES tumor testes (**f**). The relative expression level was represented as the fold difference to the value of control taking as 1 and shown as the mean ± S.E. from at least four independent experiments performed on age matched vehicle-treated control and DES-exposed group. *P < 0.05 compared to control

These studies were carried out in RNA extracted from testes showing well-formed tumor. There were no germ cells or sperm in the testes and mostly had OCT-4 and CD166 positive CSCs and cancer cells. Thus, the results indicate epigenetic alterations in transformed VSELs into CSCs. Both imprinted Igf2-H19 (Fig. 10a) and Dlk1-Meg3 (Fig. 10b) loci were affected and increased expression of IGF2 and Dlk1 was clearly evident. Expression of various enzymes responsible for DNA methylation was also affected by neonatal exposure to DES (Fig. 10c). While Dnmt-1 and Dnmt-3L were downregulated, Dnmt-3a and Dnmt-3b were upregulated. While Dmrt1, Ezh2 and p57kip2 were downregulated, Pten showed slight, nonsignificant increase (Fig. 10d). Testicular stem cells express Erα and Erβ [9] and FSHR and FSH exert its action via Fshr3 [11]. DES treatment resulted in > 12-fold increase in ERβ (Fig. 10e), whereas ERα remained unaffected. Also, rather than the canonical Fshr1, alternately spliced Fshr3 was > 40-fold upregulated (Fig. 10f).

## Discussion

TGCT are the most common cancers in young men and as discussed by Rajpert-De Meyts [19] are commonly found associated with infertility, poor semen quality and testicular atrophy. Testicular atrophy, loss of seminiferous tubules, markedly reduced sperm in the epididymis and germ cells sloughing was clearly evident in adult mice testes with cancer-like changes as a result of neonatal exposure to DES in the present study (Table 1). The testes histo-architecture was disrupted but nuclear and cytoplasmic OCT-4, SSEA-1 and SCA-1 positive stem cells were scattered throughout the tumor tissue sections. Expression of c-KIT suggestive of differentiation of stem cells into spermatogonia was markedly reduced in the tumor sections. We have earlier reported that neonatal exposure to DES induces a meiotic block [13]. Thus, blocked differentiation and expansion of the stem cells compartment leads to initiation of testicular cancer. To conclude, a mouse model for T2GCT has been developed in the present study wherein 65% of mice neonatally exposed to DES exhibit various hallmark features of T2GCT during adult life (D100) and are summarized in Table 1. This is an important advance since Spiller and Bowles [2] pointed out that a mouse model for T2GCT is not yet available. This advance will help better understand the etiology of T2GCT and also to evolve better treatment options. DES exposure is related to increased cancer incidence in humans. Our results have clinical relevance since Hom et al. [8] reported a three-fold increase in testicular cancer risk among men who were exposed in utero to DES, implicating that similar stem cell-related mechanisms may exist to explain etiology of testicular cancer in men. Neonatal acute leukemia was observed in an infant whose mother was exposed to DES in utero [28]. Multiple organs are at a risk to endocrine disruption since similar VSELs expressing steroid hormone receptors exist in multiple organs in the body including the hematopoietic system. Mierzejewska et al. [29] have earlier reported sex hormone receptors on VSELs from the hematopoietic system and ten days of estrogen treatment resulted in increase in VSELs from ~ 2% to ~ 15–40%. The results provide scope for further research and more research needs to be undertaken to ensure a paradigm shift in the current understanding.

**Table 1:** Various features of T2GCT observed in mice neonatally exposed to DES

1	Disrupted spermatogenesis, increased inflammation, giant cells with vacuolated cytoplasm (GCNIS) were observed in big sized testes with increased vascularity
2	Increased expression of OCT-4. Despite atrophied appearance of the testes with loss of seminiferous tubules, increased numbers of OCT-4 positive (both nuclear and cytoplasmic) cells were observed. This was associated with reduced expression of spermatogonial marker c-KIT and MVH
3	Testicular cancer cells had high proliferative potential as evident by increased expression of PCNA and Ki-67 and proliferation occurred in a Sertoli cells independent manner since SOX9 expression was affected by DES treatment
4	Global hypomethylation was evident due to low or reduced expression of 5-methyl cytosine and altered expression of DNMTs
5	Disrupted expression of chromatin modulator NP95 and tumor suppressor p53 [13], PTEN and Meg3
6	Increased expression of growth promoting imprinted gene Igf2 and Dlk-1
7	Down regulation of cyclin dependent kinase inhibitor p57KIP2
8	Reduced expression of H19 and Meg3 which negatively affects cell proliferation
9	Increased expression of CD166, a marker for cancer stem cells. CD166 positive CSCs appeared to be shedding from surface into the peritoneal cavity
10	The tumor-like growth was associated with increased Erβ and FSHR3

Heaney’s group reported that loss of NANOS2 expression in the fetal testicular germ cells is associated with failure to enter mitotic arrest, retention of pluripotent markers and delayed differentiation into germ cells [30]. Single-cell RNAseq studies on NANOS2 deficient germ cells (collected on D15.5) and embryonal carcinoma cells showed that both develop a transcriptional profile enriched for MYC and NODAL signaling and primed pluripotency. Also, that embryonal carcinoma arises from NANOS2 negative germ cells. Failure to initiate male sex-specific differentiation program transforms the germ cells and this is a prerequisite for TGCT initiation. This group thus provided a mechanism how fetal defects during early embryonic development may lead to testicular cancer in adult life. However, besides fetal, even the insults during neonatal life (when NANOS positive germ cells have already developed normally) could lead to reduced sperm counts, infertility and testicular cancers in adult life. Defective spermatogenesis is observed during adult life and it needs to be understood that germ cells have a limited life span and are continuously replaced. Thus, the model proposed [30] fails to explain how neonatal insults to the germ cells could manifest disease state in adult life. They have rather shown an association suggesting that germ cells are defective in T2GCT in agreement with our hypothesis that testicular cancers initiate due to expansion of stem cells compartment and blocked differentiation (spermatogenesis). We concluded this based on our observations that neonatal exposure to DES leads to sevenfold increase in VSELs numbers and almost fivefold reduction in c-Kit positive spermatogonial cells along with decreased 4n pachytene spermatocytes compared to age-matched vehicle-treated control. Indeed, it is the primitive, tissue-resident VSELs that get transformed into CD166 positive cancer stem cells and exhibit epigenetic changes (discussed below) resulting in T2GCT. Transcriptome study of purified population of primitive VSELs from control and tumor tissue will provide deeper understanding how tissue-resident VSELs get transformed into cancer stem cells.

Transition from c-Kit negative to positive spermatogonial cells is governed by an epigenetic switch which decides whether the cells undergo self-renewal or cross the point of no return (lose stemness) and initiate differentiation [30]. This is regulated by DNA methylation machinery and NP95. DNA methylation is essential for normal development and plays important role in the regulation of gene expression, genomic stability and imprinting. DNA methylation is post-replicative process and is controlled by the DNA (cytosine-5) methyltransferases (DNMTs). Undifferentiated embryonic stem cells highly express Dnmts and Dnmt-deficient ESCs die during differentiation suggesting, that Dnmts play a crucial role in maintaining pluripotency and differentiation of ESCs [32]. Similarly, VSELs show increased expression of all Dnmts and are highly enriched for Dnmt3L whereas the somatic cells exhibit reduced expression of de novo Dnmts and Dnmt3L under normal conditions [18]. NP95 plays a pivotal role in maintaining genome-wide global DNA methylation by recruiting Dnmt1 to hemi-methylated sites at replication fork in dividing cells and PCNA was also involved in this process [33, 34]. Expression of Dnmts was found disrupted in the adult testes upon neonatal expression of DES in the present study. Both Dnmt-3a and Dnmt-3b were up-regulated and Dnmt-1 was down-regulated. Earlier we have reported disruption of NP95 expression on D100 after neonatal DES exposure [13]. However, increased proliferation activity of the stem cells in the testicular sections was evidenced based on increased expression of both PCNA and Ki67 compared to age-matched vehicle-treated controls.

The epigenetic equilibrium of the testicular cells was dramatically disturbed during tumorigenesis after neonatal exposure to DES and resulted in global hypomethylation due to disrupted NP95, reduced 5-mC in combination with increased proliferative rate. Similar global hypomethylation is reported in the undifferentiated germ cell tumors including T2GCT [35]. Loss of imprinting at IGF2-H19 locus resulted in increased expression of IGF2 and reduced H19 which possibly pushed the VSELs out of their quiescence resulting in their excessive self-renewal. These epigenetic alterations convert quiescent VSELs in normal tissues into actively dividing CSCs in the T2GCT. As a result, increased expression of OCT-4, SSEA-1 and SCA-1 was observed in DES-treated tumor sections but their further differentiation into cKit positive spermatogonia was blocked. Similarly, higher Dlk1-Meg3 expression ratio in DES-treated testes suggested a possible role in malignant growth. It is suggested that overexpression of Dlk1 enhances tumor cell stemness and invasiveness in-vitro [36, 37]. Meg3 encodes an lncRNA which functions as a tumor suppressor and serves as a surrogate marker of downstream proliferation-inhibiting miRNAs [38]. Yang et al. [39] suggested that Meg3 regulates tumorigenesis through interaction with PTEN/PI3k/Akt signaling pathway in TGCT. Loss or significant reduction in Meg3 expression is found in primary tumors. Here we also found suppressed Meg3 upon DES exposure which suggestive of contribution of Meg3 in tumor development.

PTEN is a tumor suppressor gene and was minimally expressed in DES-treated testicular sections but the transcripts were increased, although not significantly. This contradiction was intriguing. Vizio et al. [40] reported complete lack of PTEN in the seminomas, carcinomas and teratomas and a fraction of germ cell tumors are known to retain PTEN mRNA despite reduced PTEN protein expression, suggestive of involvement of some post-transcriptional mechanism. Embryos deficient in Ezh2 fail to develop and do not allow ES cell lines to be derived [41]. It has an important role during histone modifications which are crucial to ensure epigenetic changes. It adds trimethylation group to histone H3 at lysine 27 and this leads to gene inhibition. Stem cell self-renewal is not affected in Ezh2 deficient mice but their differentiation is affected and cells show elevated levels of Oct-4 and Nanog [41]. Ezh2 expression was reduced upon DES treatment associated with increased expression of pluripotent markers and blocked differentiation.

p57KIP2 gene, which encodes a cyclin-dependent kinase inhibitor, undergoes genomic imprinting and lies within a 700-kb domain of imprinted genes including IGF2 and H19. Loss of imprinting of this region is associated with embryonal malignancies. It regulates the cell cycle and is frequently down-regulated in malignancies through several mechanisms, denoting its anti-oncogenic function [42]. VSELs show increased expression of p57KIP2, a known negative regulator of the cell cycle and plays an important role in maintaining VSELs in a quiescent state [18]. Reduced expression of p57kip2 was observed in DES-treated testicular sections and possibly support active self-renewal of VSELs in testicular tumors.

Epigenetic modifications contribute to genetic instability and to the neoplastic process. Testicular tumors are considered to be estrogen-dependent cancers. Dumasia et al. [43] reported that endocrine disruptors signal through ERβ to induce epigenetic disturbances by regulating DNA methylation and its machinery in the adult rat testes. Almost 12-fold increase in ERβ was detected in the present study, whereas Erα remained minimally expressed. FSHR has been reported in several types of cancers including testicular cancer [44, 45]. Functional FSHR expression is also reported on testicular VSELs and SSCs [11]. FSHR expressing VSELs survive in the chemoablated testes [10, 11] and FSH treatment resulted in increased numbers of stem cells. This effect of FSH is mediated via Fshr3 and not through canonical Fshr1 [11]. DES-treated testis with tumor-like changes showed > 40-fold up-regulation of Fshr3 whereas Fshr1 remained minimally expressed.

Stem cells get affected by endocrine disruptors but why testicular tumor was observed only in DES-treated mice and not reported when mice are exposed to other endocrine disruptors including estradiol (20 µg/pup/day on days 5–7) which was studied by us in an earlier study [13]. Endocrine disruptors lead to varied range of responses depending on the extent of disruption [46] described as testicular dysgenesis syndrome [47, 48]. What changes lead to testicular cancer initiation was of great interest to us in the present study. In order to address this, expression of certain markers including 5-mC was studied after neonatal exposure to estradiol and the results are provided in Additional file 1: Fig. S9. Upon tabulating all the markers and comparing the varied effects after neonatal exposure to DES and E2 (Additional file 1: Table S5), crucial changes that initiate testicular cancer include increased numbers of transformed VSELs expressing embryonic markers and CD166 accompanied by blocked differentiation, loss of Sertoli cells, global hypomethylation, loss of p53 and Pten along with increased expression of Ki67. Interestingly, exposure to estradiol led to increased PCNA but not Ki67 whereas after DES treatment Ki67 was several folds increased suggesting marked hyperplasia in the cancer sample. Such an environment of greatly increased Ki67 expression and genomic instability could result in somatic mutations.

To conclude, DES induces epigenetic changes which push the testicular VSELs to come out of their quiescence and undergo excessive self-renewal along with significant block of their differentiation as discussed earlier [16]. Evidence is provided in the present study to support the notion that it is the tissue-resident VSELs that get transformed into CSCs to initiate testicular cancer. These CSCs (transformed VSELs) express pluripotent markers and exist in adult testes where they initiate testicular cancer. The existing concept of fetal survival of GCNIS needs to be re-examined as was discussed earlier [14]. Embryonic markers including OCT-4 are expressed in cancers arising in multiple adult tissues [24, 25, 49] and thus specifically testicular cancers having fetal origin as a pre-CIS does not make sense. The concept of pre-CIS was put forth to explain expression of pluripotent markers in testicular cancer cells but the presence of pluripotent VSELs in adult testes provides logical explanation for expression of embryonic markers in not only testicular but tumors arising in multiple organs and was discussed earlier by us [14].

## Conclusions

The results of the present study show that exposure of mice pups to diethylstilbestrol blocks spermatogenesis and transform OCT-4 positive VSELs into putative cancer stem cells that increase in numbers, are epigenetically distinct and co-express OCT-4 and CD166. The results shed a new light on pathogenesis on Type II germ cell tumors and also it demonstrates for the first time that quiescent VSELs are implicated in initiation of testicular cancer.

### Supplementary Information

**Additional file 1.** Supplementary data including 4 Suppl Tables and 9 Suppl Figs.

## Data Availability

This is provided in the supplement.
