# Correction: Testicular cancer in mice: interplay between stem cells and endocrine insults

**DOI:** 10.1186/s13287-023-03510-5

**Published:** 2023-09-27

**Authors:** Ankita Kaushik, Deepa Bhartiya

**Affiliations:** Stem Cell Biology Department, ICMR-National Institute for Research in Reproductive and Child Health, Jehangir Merwanji Street, Parel, Mumbai, 400 012 India

**Correction: Stem Cell Research & Therapy (2022) 13:243** 10.1186/s13287-022-02784-5

Following the publication of our article [1], readers noted that one Figure was placed at two places (Figs. 3d and 6e) and also there was partial overlap between Fig. 3g and 6d. Figure 3d was placed incorrectly due to unintentional human error. Figures 3g and 6d represent same experimental conditions and observations. The corrections have now been incorporated in Figs. 3d and 3g; Fig. 3 has been modified and given below with modified (underlined) legend while Fig. 6 remains unchanged. The changes do not change the results and conclusions of the original study.

**Fig. 3:**
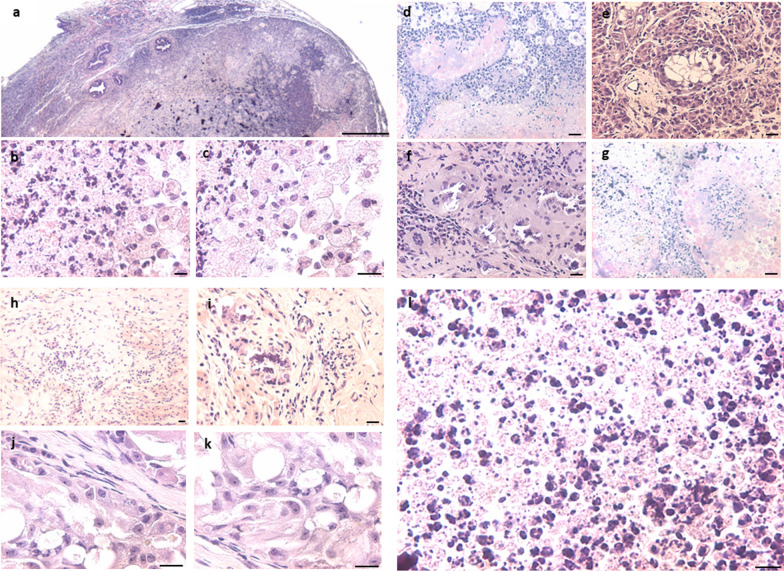
Neonatal exposure to DES resulted in bigger size testes on day 100. Bigger testis showed completely disrupted histo-architecture (**a**). Seminiferous tubules were markedly reduced, occluded, and without a defined membrane, and germ cells were depleted (**d**, **e**, **g**). Sertoli cells were also not observed (**d**–**k**). Massive inflammation was observed in testes (**i**). Degeneration of germ cells was observed. GCNIS-like stem cells were clearly observed restricted to small seminiferous tubules (**d**, **e**, **f**, **g**). Small, spherical putative stem cells were present among the inflammatory cells (**h**, **i**, **l**). Multinuclear giant cells were observed (**b**, **c**). Scale: (a:100 μm, k-l: 20 μm)
