# Supplementary material for: Testicular cancer in mice: interplay between stem cells and endocrine insults
Source: Stem Cell Res Ther. 2022 Jun 8;13:243. doi: 10.1186/s13287-022-02784-5 (PMC9175365; doi:10.1186/s13287-022-02784-5)
Supplement: Supplementary file 1 — Additional file 1. Supplementary data including 4 Suppl Tables and 9 Suppl Figs. [file 13287_2022_2784_MOESM1_ESM.pdf]

Supplementary section

Testicular cancer in mice: Interplay between stem cells and endocrine insults

Ankita Kaushik and Deepa Bhartiya. Stem Cell Biology Department, ICMR- National Institute for Research in Reproductive Health, Jehangir Merwanji Street, Mumbai, 400 012 India.

T **Suppl Table 1**

**5-methyl cytosine**	Global methylation status was studied by 5 methyl cytosine (5mC) expression and gave a fair idea whether DES treatment affects DNA methylation. Dumasia et al [1] reported significant global hypomethylation regulated by estrogen signaling through Erβ. Global DNA hypomethylation is associated with genomic instability and cancer predisposition.<br><br>Hypomethylation confers a tumor-suppressing phenotype
**IGF2-H19**	H19 and Igf2 genes are part of a cluster of imprinted genes.<br><br>VSELs show erasure or hypomethylation of imprints in paternally methylated and hypermethylation of imprints in maternally methylated ones. These epigenetic characteristics result in upregulation of H19 and repression of Igf2 in VSELs and thus they remain quiescent [2].<br><br>This balance may be affected by endocrine disruption and explain increased numbers of VSELs after neonatal exposure to DES.<br><br>Boissonnas et al [3] published specific epigenetic changes in IGF2-H19 locus in infertile men.<br><br>Pathak et al [4] have reported reduced DNA methylation at Igf2-H19 ICR in sperm upon 60 days of tamoxifen (0.4 mg/kg body weight/day orally, 5 days a week) treatment to 75 days old rats which upon mating resulted in embryo loss.<br><br>Doshi et al [5] reported aberrant DNA methylation at Igf2–H19 imprinting control region in sperm upon neonatal exposure to bisphenol A and its association with post implantation loss.<br><br>Khambata et al [6] reported similar altered methylation status of several imprinted genes in male partners from couples experiencing recurrent pregnancy loss.
**Dlk1-Meg3**	Maternally Expressed Gene 3 (Meg3) is an imprinted DLK1–MEG3 locus gene. It encodes a long, non-coding RNA that has been shown to regulate tumorigenesis through interaction with PTEN/PI3k/Akt signaling pathway in TGCT [7].<br><br>MEG3 encodes a lncRNA which is suggested to function as a tumor suppressor and has been involved in a variety of cancers.<br>Zheng et al [8] demonstrated that MEG3 acts as a tumor suppressor<br><br>Increased expression of DLK1 is found in several types of cancers. Overexpression of Dlk1 enhances tumor cell stemness & invasiveness in-vitro [9,10].
**Ezh2**	Ezh2 has an important role during histone modifications which are crucial to ensure epigenetic changes. It adds trimethylation group to histone H3 at lysine 27 and this leads to gene inhibition.<br><br>Stem cell self-renewal is not affected in Ezh2 deficient mice but their differentiation is affected and cells show elevated levels of Oct-4 and Nanog [11].

	Interestingly, in Ezh2 plays an essential role in the maintenance of both the proliferative and self-renewal capacity of stem/progenitor cells and the full execution of their differentiation.
**DNA methylation enzymes**	DNA methylation, catalyzed by the DNMTs, plays an important role in maintaining genome stability.<br><br>Altered expression of DNMTs and disruption of DNA methylation patterns are closely associated with many forms of cancer.<br><br>Dnmt 1 is involved in the maintenance of DNA methylation, Dnmt 3 a & b for de novo methylation, Dnmt 3L shares homology with Dnmt 3 family & essential for DNA methylation.<br><br>BPA treated testis showed increased levels of Dnmt3a and b [5].
**PTEN**	PTEN is a tumor suppressor gene that is frequently mutated or deleted in sporadic human tumors.<br><br>PTEN negatively regulates cell growth, migration and survival via the phosphatidylinositol 30-kinase/AKT signaling pathway.<br><br>PTEN is expressed in germ cells and is virtually absent from 56% of seminomas, 86% of embryonal carcinomas and virtually all teratomas [12].<br><br>Loss of PTEN marks the transition from intra-tubular germ cell neoplasia (ITGCN) to invasive germ cell tumors [12].
**p57kip2**	Cyclin-dependent kinase inhibitor 1C (p57(KIP2) is an imprinted gene that regulates cell cycle and is frequently down-regulated in malignancies denoting its anti-oncogenic function.
**OCT-4A**<br>**OCT-4**	VSELs are pluripotent stem cells and express nuclear OCT-4 whereas the immediate descendants where differentiation is initiated express cytoplasmic OCT-4. VSELs with nuclear OCT-4A and SSCs with cytoplasmic OCT-4 are expressed in both human [13] and mice testes [14-16].<br><br>OCT4 is a sensitive and specific biomarker for intratubular germ cell neoplasia of the testis [17-18].
**SCA-1**	Stem cell antigen 1 (SCA-1) is expressed on stem/progenitor cells in multiple organs and is also reported in testes [16].
**SSEA-1**	Cell surface stage-specific embryonic antigen 1 (SSEA-1) is a specific marker for pluripotent stem cells including VSELs in mice [19].
**c-KIT**	Epigenetic status of spermatogonia changes dramatically during transition from c- Kit negative to positive state and this epigenetic switch decides whether the cells undergo self-renewal or cross the point of no return (lose stemness) and initiate differentiation [20].<br><br>C-kit is a proto-oncogene and KIT mutations are common in testicular seminomas [21].
**Dmrt1a**	Dmrt1 is a regulator of testis development and is implicated in testicular germ cell tumors of mouse and human. Loss of Dmrt1 in 129Sv strain mice results in a >90% incidence of testicular teratomas [22].<br><br>DMRT1 is a transcription factor, belonging to DNA binding gene family and is expressed in TGCTs in addition to Oct-4 and NANOG [23].
**PCNA**	Proliferating cell nuclear antigen (PCNA) is well established marker specific to DNA synthesis in dividing cells.
**Ki67**	Ki67 is a marker for proliferation. Serves as a molecular target in the diagnosis of cancer [24].
**CD166**	It is a cell surface marker for stem/progenitor cells in several cancers including testicular cancer in both mice and men [19, 25,26].

Suppl Table 2

List of antibodies used in the study

Antibodies	Dilution	Company
OCT-4	1:100	Abcam, Cambridge ab18976
OCT-4A	1:100	Millipore MAB4401
SSEA-1	1:100	Abcam
SCA-1	1:100	Biolegend 122501
MVH	1:100	Abcam Ab13840
c-KIT	1:100	Millipore
SOX-9	1:100	Abcam, Cambridge ab185230
PCNA	1:200	Sigma Aldrich P8825
Ki-67	1:100	Abclonal A2094
PTEN	1:100	Abcam, Cambridge ab32199
5-methyl cytosine (5-mC)	1:100	Abclonal A2341
ALCAM/CD166	1:200	R&D SYSTEMS BAF1172

Suppl Table 3

Primer sequences for various transcripts used in the study

Genes	Sequence (5'-3')	Annealing
IGF-2	F: GTGTGTGTCAGCCAAGCATG<br>R: CAATGTGGGGACACAGAGG	58
H-19	F: TGCTCCAAGGTGAAGCTGAAAG<br>R: GCAGAGTTGGCCATGAAGATG	62
DLK-1	F: CCCGGCCATCTGCTTCAC<br>R: CGCCGCTGTTATACTGCAAC	58
MEG-3	F: CAGAGCGCTTCTGAAGACCA<br>R: CACCTACTGGGTGCTCACTG	58
PTEN	F: TTCATACCAGGACCAGAGGA<br>R: TTGTCATTATCTGCACGCTCT	60
p57Kcip2	F: GCGCAAACGTCTGAGATGAG<br>R: CCATCTCCGGTTCCTGCTAC	59
EZH-2	F: CAGTTTCGTCTTCCACCATAAA<br>R: CAGCACAAGTCATCCCGTTAA	60
DNMT-1	F: CTGCAAGGACATGAGCCCAC<br>R: CCTGTATGTTGGGCAGGTCAC	62
DNMT-3a	F: GAGGCAGTCCCTGCAATGAC<br>R: GCGGCCAGTACCCTCATAAAG	62
DNMT-3b	F: TGTGCAGAGTCCATTGCTGTAGGA<br>R: GCTTCCGCCAATCACCAAGTCAAA	62
DNMT-3l	F: GCTGAAGAGCAAGCATGCG<br>R: TCTTCACCAGGAGGTCAACTTTC	62
ERα	F: CCTTCTAGACCCTTCAGTGAAGC<br>R: CGAGACCAATCATCAGAATCTCC	62
ERβ	F: GGGTGAAGGAGCTACTGCTG<br>R: CCAATCATGTGCACCAGTTC	58
Fshr1	F: TGGAGGCGGCAAACCTCTGAAC<br>R: TCTGGCTTTGGCGAGCAGGTC	65
Fshr3	F: TGTCCCTAAGGACTTGCACTG<br>R: AATGCTCTGCCTGTCACTGTC	56
18S	F: GGAGAGGGAGCCTGAGAAAC<br>R: CCTCCAATGGATCCTCGTTA	62

Suppl Table 4

Total number of mice studied

No. of mice	Total number of mice studied<br><br>70 mice on D100 after DES
28	Bigger testes with bilateral tumor-like growth<br><br>Testes were reddish & epididymis adhered to testes
18	Pale yellow big testes, hard to touch
15	One bigger and one atrophied
5	3 with rudimentary testes and 2 mice testes lost
4	Both testes as in normal but with altered spermatogenesis
Total number of aged mice studied: 12<br><br>• 6- both testes enlarged<br>• 4- one big one smaller<br>• 2- both tetes atrophied	
14 mice died but cause of death could not be ascertained<br><br>Extra-testicular growth was observed in one D100 mouse	

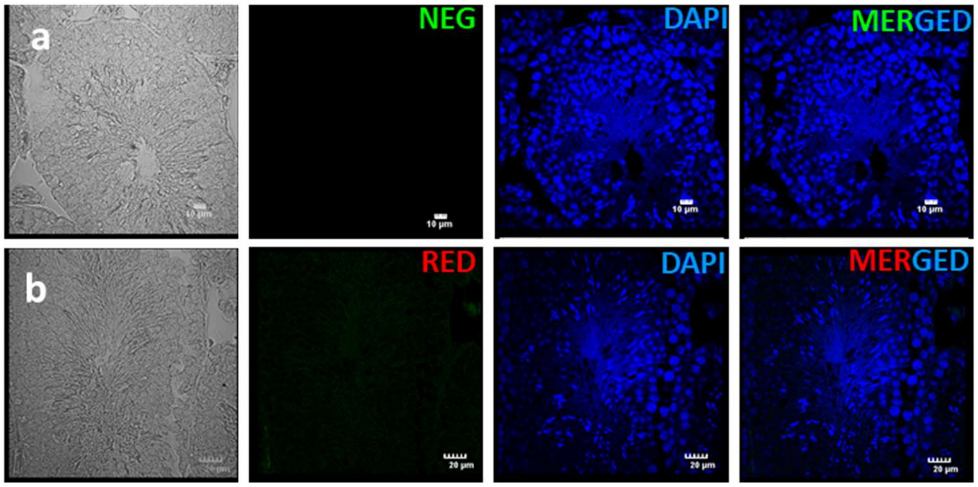

Suppl Fig 1. Negative controls. Negative controls used in study with omission of primary antibody.

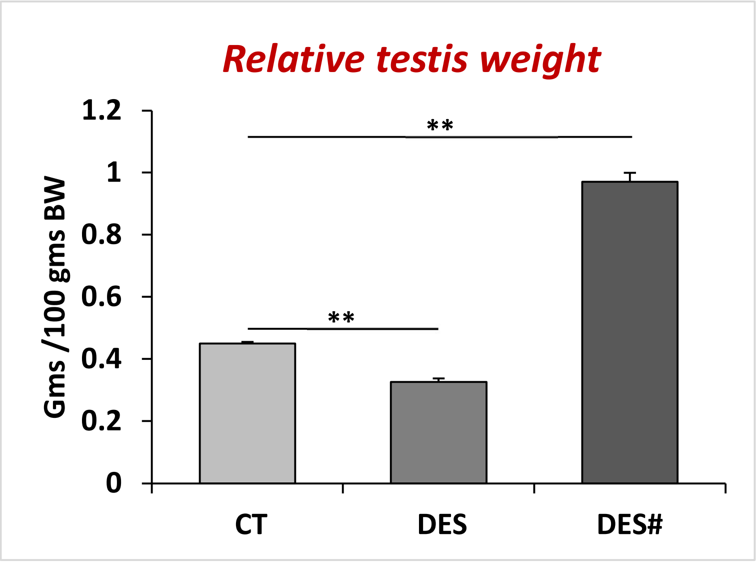

Suppl Fig 2: Relative testis weight upon neonatal DES exposure compared with age-matched vehicle treated control at D100. Neonatal DES exposure resulted in reduced relative testicular weight however there are mice with testicular tumor-like growth has significant increased relative testis weight (DES#). **$p<0.01$ compared to control.

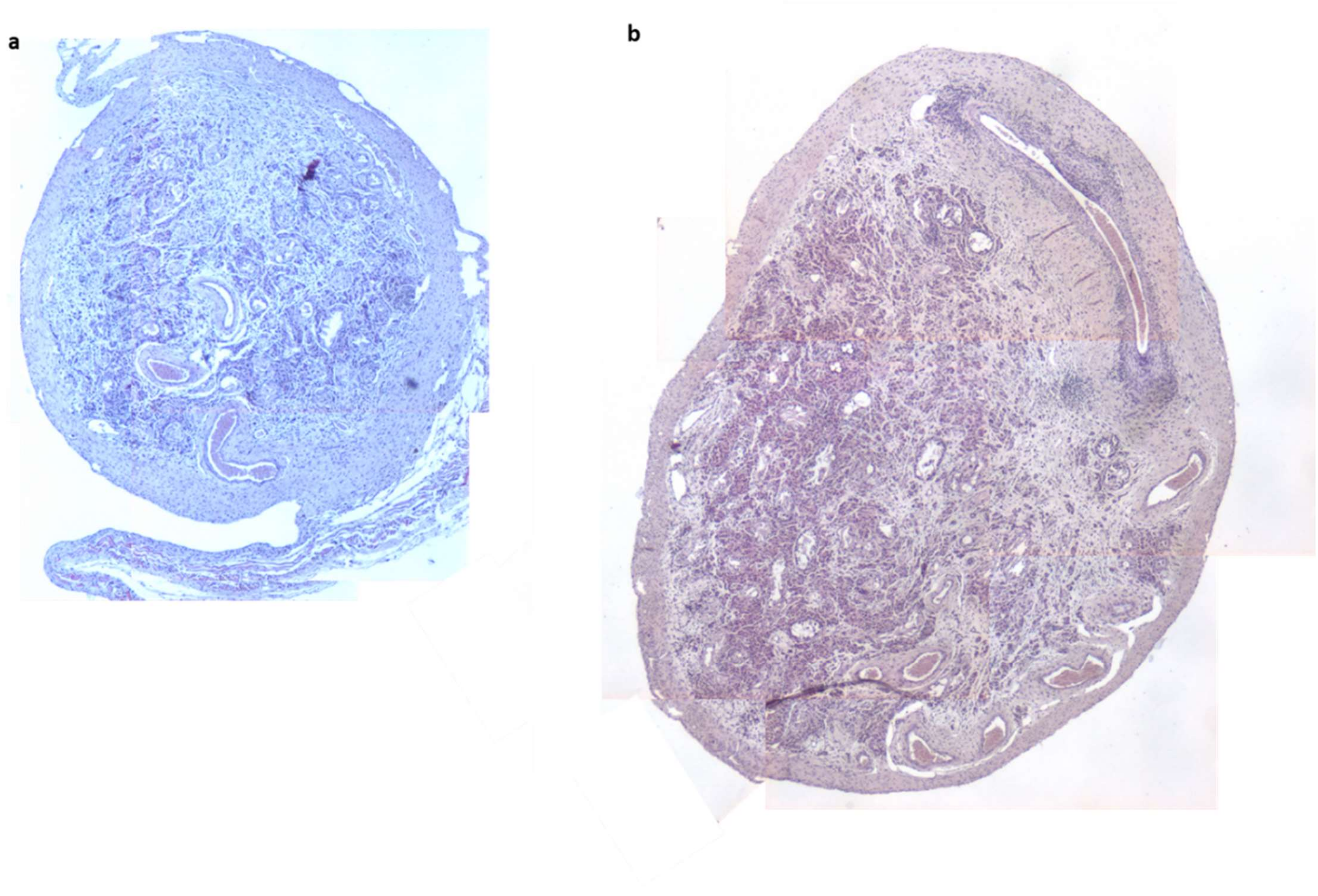

Suppl Fig 3: Histological sections of DES exposed testes. Images of testes captured with a microscope and then stitched together to show complete cross sections. Testicular shape was change with complete loss of histoarchitecture.

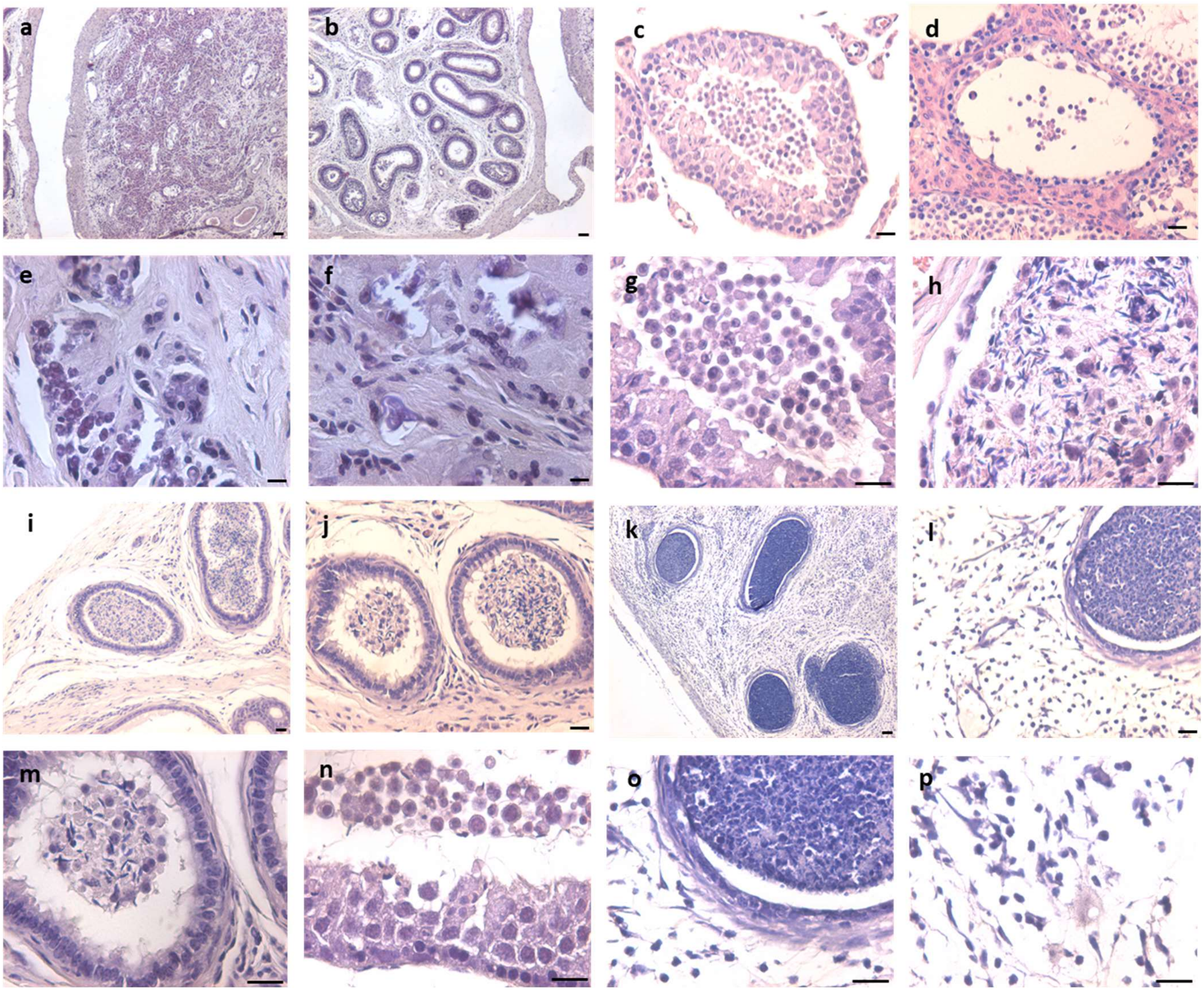

Suppl Fig 4. DES treatment testis of bigger size. Testicular tissue lacked tubules (a-b) but there is increased cellularity. Epididymis was devoid of sperm and multi-layered (i-l). At places undifferentiated atypical germ-like cells were present (c-h,o-p). The epididymis lumen also had undifferentiated pluripotent cells along with sperm at places (m-n) whereas at places lumen was full of inflammatory cells. Scale bar 20µm.

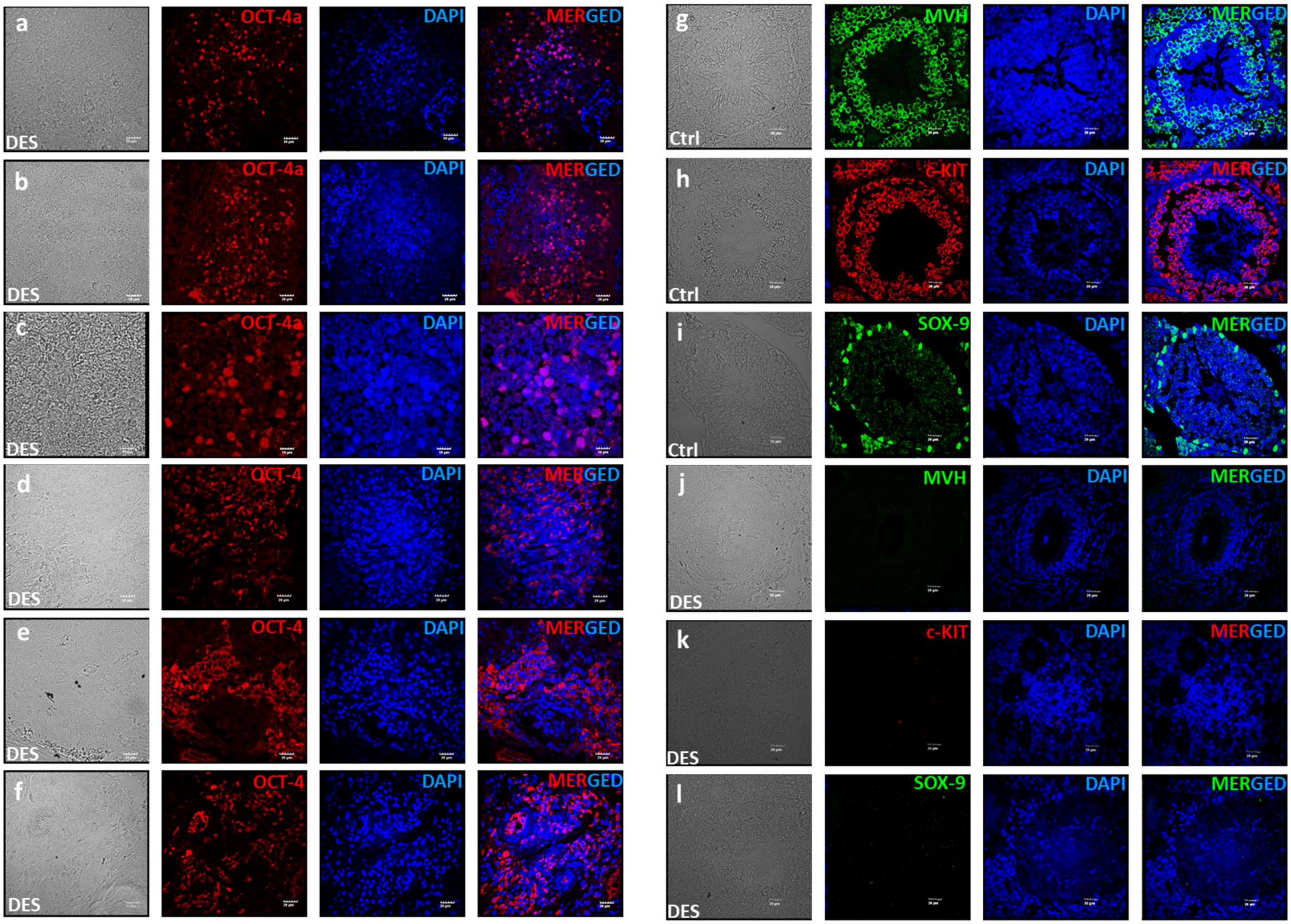

Suppl Fig 5. Immunostaining of VSELs specific OCT-4A, OCT-4, MVH, c-KIT and SOX-9 proteins in DES testicular paraffin section compared with age-matched vehicle treated control. Note increased expression of VSELs specific nuclear OCT-4A (a-c) and cytoplasmic OCT-4 (d-f) after endocrine disruption with DES. Control mice expressed germ cell specific marker **(**MVH, g), spermatogonial marker (c-KIT, h), and Sertoli cells specific marker (SOX-9, i) whose expression was lost after DES treatment (j-l, respectively)

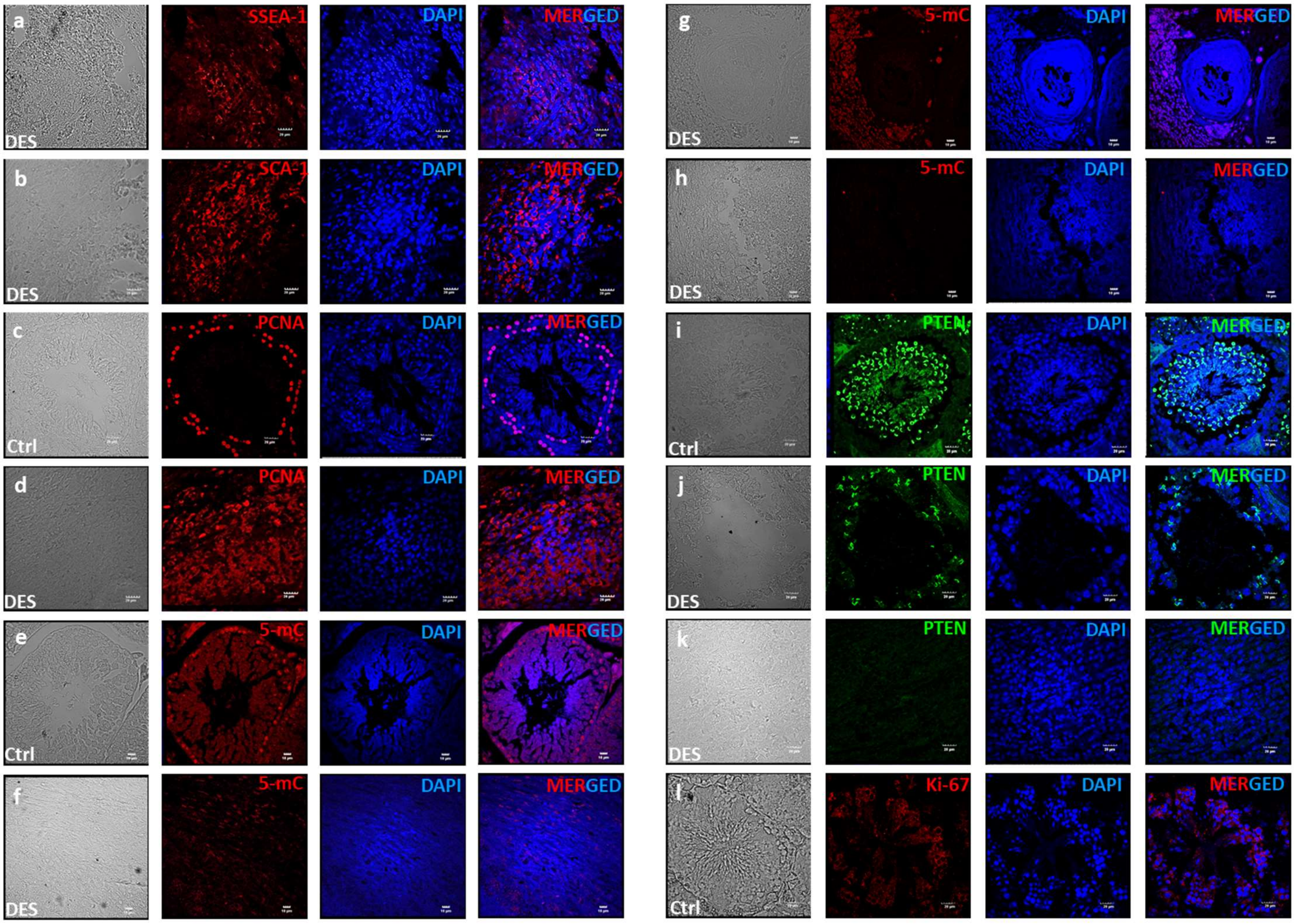

Suppl Fig 6. Immuno-localization of SSEA-1, SCA-1, PCNA, Ki-67, PTEN and Ki-67 in DES testicular paraffin section compared with age-matched vehicle treated control. Note increased expression of pluripotent marker (SSEA-1, a) and Stem Cell Antigen (SCA-1, b) upon DES treatment. DES treated testes showed increased PCNA (d) expression compared with normal testicular section (c). Global hypo-methylation was observed after DES endocrine disruption (f-h) while control testicular section has normal methylation (e). Tumor suppressor PTEN was abundantly expressed by normal testicular germ cells (i) and DES testis showed lost of PTEN expression (k) however (j) in atrophied testis few germ cells expressed PTEN was observed. (l) shows minimal expression of Ki-67 staining in normal control testis section.

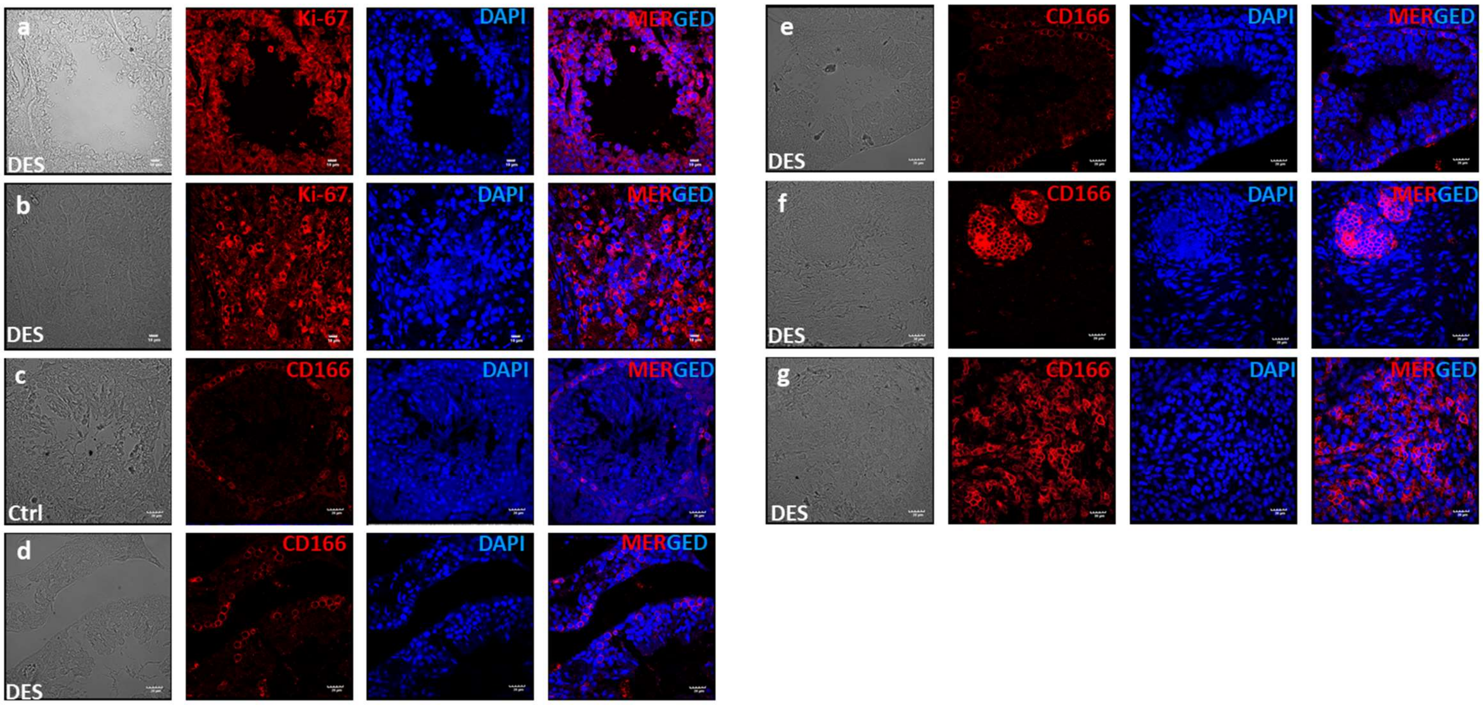

Suppl Fig 7. Immuno-localization of Ki-67 and CD166 in DES testicular paraffin section compared with age-matched vehicle treated control. Ki-67 expression (a-b) and CD166 (f-h) was massively increased upon DES treatment, conversely vehicle treated normal testis (c) and atrophied testis (e-d) has minimal expression of CD166.

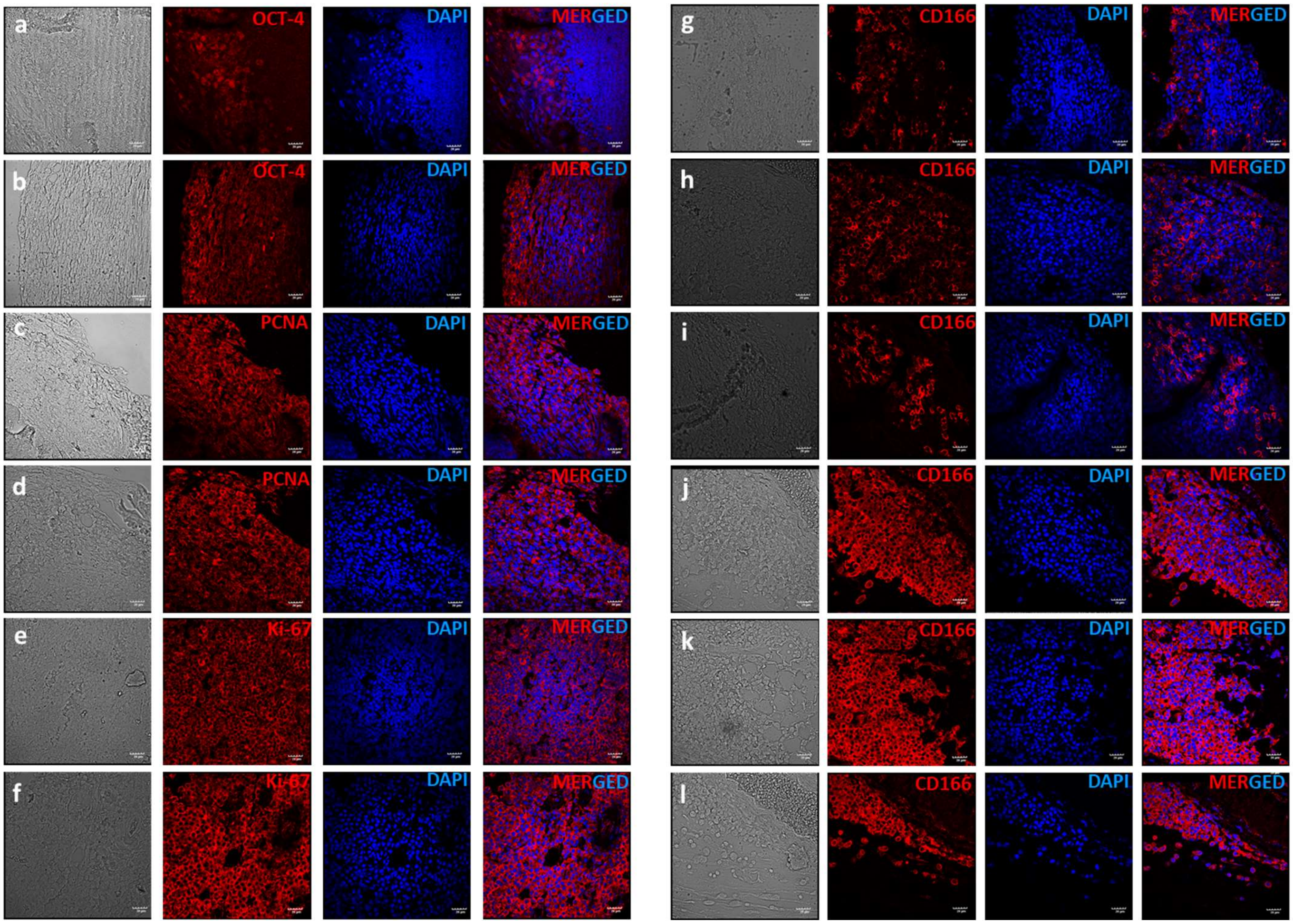

Suppl Fig 8. Immuno-localization of OCT-4, PCNA, Ki-67 and CD166 in extragonadal tissue. Increased expression of OCT-4 (a-b), PCNA (c-d), Ki-67 (e-f) and CD166 (g-l) was observed in extragonadal tissue.

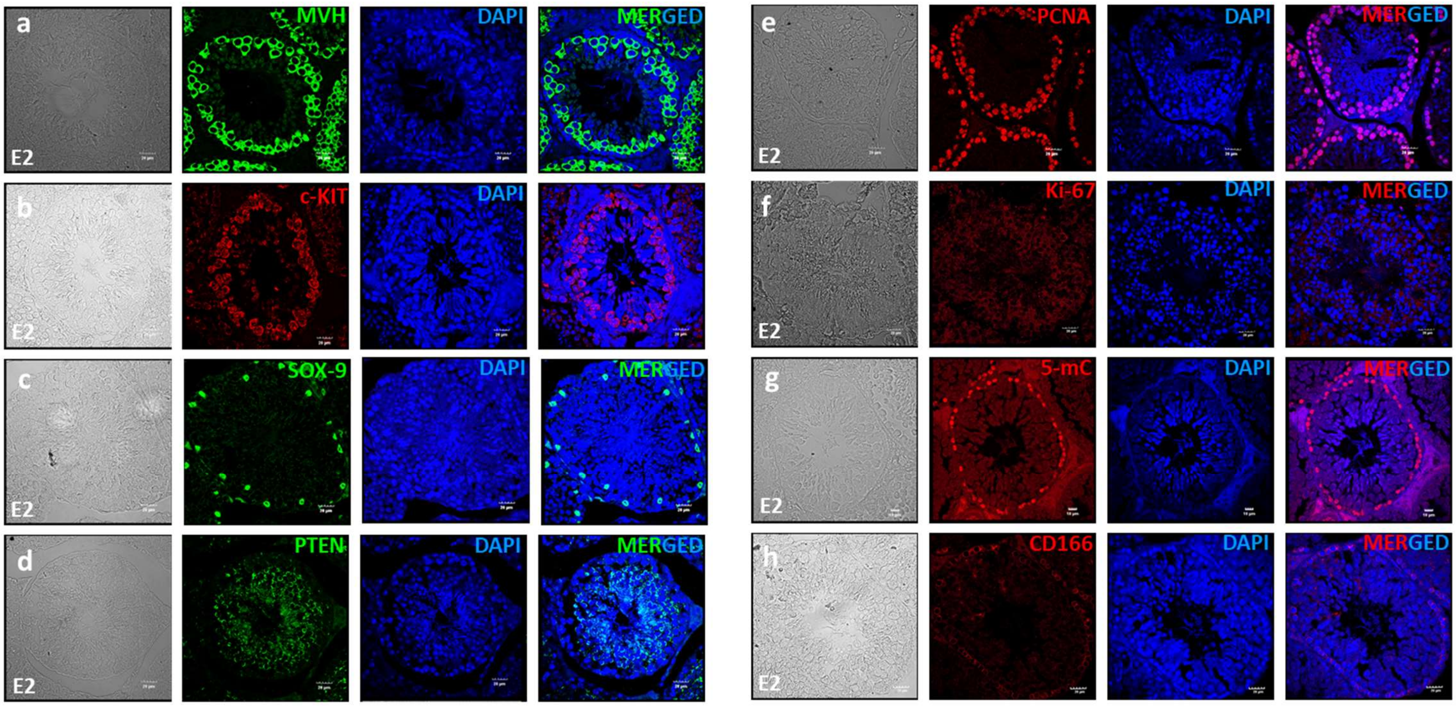

Suppl Fig 9. Immuno-localization of MVH, c-KIT, SOX-9, PTEN, PCNA, Ki-67, 5-mC and CD166 in E2 treated testicular paraffin section on D100. MVH (a), PTEN (d) and SOX-9 (c) were not affected. c-KIT positive cells were observed (b) but to a lesser extent from normal age-matched control (Suppl Fig 2, h) Global methylation pattern was normal as evident by expression of 5-mC (g). PCNA was increased (e) but Ki67 was not increased (f). It is noteworthy that CD166 was not expressed (h). We did not observe tumor-lie changes upon E2 exposure at D100.

Suppl table 5

Molecular changes induced by DES and E2 on adult mice testes

	DES Treatment	***Estradiol treatment***
**VSELs numbers**	7 folds increase compared to ctrl	2 folds increase compared to ctrl
**Embryonic markers OCT-4, SCA-1, SSEA-1**	Highly expressed<br>Oct4a >8 fold by q-RTPCR	Slightly Increased<br>Oct4a >3 fold by q-RTPCR
**Germ cells, MVH, c-KIT**	Completely blocked differentiation	Still observed
**Sertoli cells SOX9**	Complete loss	Still observed
**Global hypomethylation**	5mC was not detected	Expressed
**Tumor suppressor genes PTEN, P53**	Loss of tumor suppressor	Not affected
**PCNA, Ki67**	PCNA & Ki67 massively expressed	PCNA increased but minimal Ki67
**Igf2-H19<br>Dlk- Meg3**	Highly Increased Igf2& Dlk<br>Suppressed H19 & Meg3	Noticeably increase Igf2& Dlk,<br>Suppressed H19 & Meg3
**Dnmts**	Altered methylation machinery	Altered methylation machinery
**CD166**	Increased expression	Minimally expressed

References

1. Dumasia K, Kumar A, Deshpande S, Balasinor NH. Estrogen signaling, through estrogen receptor β, regulates DNA methylation and its machinery in male germ line in adult rats. Epigenetics. 2017;12(6):476-483.

2. Shin DM, Zuba-Surma EK, Wu W, Ratajczak J, Wysoczynski M, Ratajczak MZ, et al. Novel epigenetic mechanisms that control pluripotency and quiescence of adult bone marrow-derived Oct4(+) very small embryonic-like stem cells. Leukemia. 2009;23(11):2042–2051.

3. Boissonnas CC, Abdalaoui HE, Haelewyn V, Fauque P, Dupont JM, Gut I, et al. Specific epigenetic alterations of IGF2-H19 locus in spermatozoa from infertile men. Eur J Hum Genet. 2010;18(1):73-80.

4. Pathak S, D'Souza R, Ankolkar M, Gaonkar R, Balasinor NH. Potential role of estrogen in regulation of the insulin-like growth factor2-H19 locus in the rat testis. Mol Cell Endocrinol. 2010;314(1):110-7.

5. Doshi T, Mehta SS, Dighe V, Balasinor N, Vanage G. Hypermethylation of estrogen receptor promoter region in adult testis of rats exposed neonatally to bisphenol A. Toxicology. 2011;289(2-3):74-82.

6. Khambata K, Raut S, Deshpande S, Mohan S, Sonawane S, Gaonkar R, et al. DNA methylation defects in spermatozoa of male partners from couples experiencing recurrent pregnancy loss. Hum Reprod. 2021;36(1):48-60.

7. Yang NQ, Luo XJ, Zhang J, Wang GM, Guo JM. Crosstalk between Meg3 and miR-1297 regulates growth of testicular germ cell tumor through PTEN/PI3K/AKT pathway. Am J Transl Res. 2016;8(2):1091-9.

8. Zheng Q, Lin Z, Xu J, Lu Y, Meng Q, Wang C, et al. Long noncoding RNA MEG3 suppresses liver cancer cells growth through inhibiting β-catenin by activating PKM2 and inactivating PTEN. Cell Death Dis. 2018;9(3):253.

9. Kim Y, Lin Q, Zelterman D, Yun Z. Hypoxia-regulated delta-like 1 homologue enhances cancer cell stemness and tumorigenicity. Cancer Res. 2009;69: 9271-9280.

10. Li L, Tan J, Zhang Y, Han N, Di X, Xiao T, et al. DLK1 promotes lung cancer cell invasion through upregulation of MMP9 expression depending on Notch signaling. PLoS One. 2014;9: e91509. doi: 10.1371/journal.pone.0091509.

11. O'Carroll D, Erhardt S, Pagani M, Barton SC, Surani MA, Jenuwein T. The polycomb-groupgene Ezh2 is required for early mouse development. Mol Cell Biol. 2001;21(13):4330-4336.

12. Di Vizio D, Cito L, Boccia A, Chieffi P, Insabato L, Pettinato G, et al. Loss of the tumor suppressor gene PTEN marks the transition from intratubular germ cell neoplasias (ITGCN) to invasive germ cell tumors. Oncogene. 2005;24(11):1882-94.

13. Bhartiya D, Kasiviswanathan S, Unni SK, Pethe P, Dhabalia JV, Patwardhan S, Tongaonkar HB. Newer insights into premeiotic development of germ cells in adult human testis using Oct-4 as a stem cell marker. J. Histochem. Cytochem. 2012;58(12):1093–1106.

14. Anand S, Bhartiya D, Sriraman K, Mallick A. Underlying mechanisms that restore spermatogenesis on transplanting healthy niche cells in busulphan treated mouse testis. Stem Cell Reviews. 2016;12:682–697.

15. Patel H, Bhartiya D. Testicular stem cells express follicle-stimulating hormone receptors and are directly modulated by FSH. Reprod Sci. 2016;11:1493–1508.

16. Kaushik A, Bhartiya D. Additional Evidence to Establish Existence of Two Stem Cell Populations Including VSELs and SSCs in Adult Mouse Testes. Stem Cell Rev Rep. 2020;16:992-1004.

17. Jones TD, Ulbright TM, Eble JN, Baldridge LA, Cheng L. OCT4 staining in testicular tumors: a sensitive and specific marker for seminoma and embryonal carcinoma. Am J Surg Pathol. 2004;28(7):935-40.

18. Rijlaarsdam MA, van Herk HA, Gillis AJ, Stoop H, Jenster G, Martens J, et al. Specific detection of OCT3/4 isoform A/B/B1 expression in solid (germ cell) tumours and cell lines: confirmation of OCT3/4 specificity for germ cell tumours. Br J Cancer. 2011; 6;105(6):854-63.

19. Zhao W, Li Y, Zhang X. Stemness-Related Markers in Cancer. Cancer Transl Med. 2017;3(3):87-95.

20. Shirakawa T, Yaman-Deveci R, Tomizawa S, Kamizato Y, Nakajima K, Sone H, et al. An epigenetic switch is crucial for spermatogonia to exit the undifferentiated state toward a Kit-positive identity. Development. 2013;140(17):3565-76.

21. Kemmer K, Corless CL, Fletcher JA, McGreevey L, Haley A, Griffith D, et al. KIT mutations are common in testicular seminomas. Am J Pathol. 2004;164(1):305-13.

22. Krentz AD, Murphy MW, Zhang T, Sarver AL, Jain S, Griswold MD, et al. Interaction between DMRT1 function and genetic background modulates signaling and pluripotency to control tumor susceptibility in the fetal germ line. Dev Biol. 2013;377(1):67-78.

23. Zhang T, Zarkower D. DMRT proteins and coordination of mammalian spermatogenesis. Stem Cell Res. 2017;24:195-202.

24. Li LT, Jiang G, Chen Q, Zheng JN. Ki67 is a promising molecular target in the diagnosis of cancer (review). Mol Med Rep. 2015;11(3):1566-72.

25. Walcher L, Kistenmacher AK, Suo H, Kitte R, Dluczek S, Strauß A, et al. Cancer Stem Cells-Origins and Biomarkers: Perspectives for Targeted Personalized Therapies. Front Immunol. 2020;7;11:1280.

26. Munro MJ, Wickremesekera SK, Peng L, Tan ST, Itinteang T. Cancer stem cells in colorectal cancer: a review. J Clin Pathol. 2018;71(2):110-116.
